# 3D Proximal Tubule Tissues Recapitulate Key Aspects of Renal Physiology to Enable Nephrotoxicity Testing

**DOI:** 10.3389/fphys.2017.00123

**Published:** 2017-03-08

**Authors:** Shelby M. King, J. William Higgins, Celina R. Nino, Timothy R. Smith, Elizabeth H. Paffenroth, Casey E. Fairbairn, Abigail Docuyanan, Vishal D. Shah, Alice E. Chen, Sharon C. Presnell, Deborah G. Nguyen

**Affiliations:** ^1^Organovo, Inc.San Diego, CA, USA; ^2^Ardea Biosciences Inc.San Diego, CA, USA

**Keywords:** proximal tubule, 3D model, nephrotoxicity, drug safety, renal transporters

## Abstract

Due to its exposure to high concentrations of xenobiotics, the kidney proximal tubule is a primary site of nephrotoxicity and resulting attrition in the drug development pipeline. Current pre-clinical methods using 2D cell cultures and animal models are unable to fully recapitulate clinical drug responses due to limited *in vitro* functional lifespan, or species-specific differences. Using Organovo's proprietary 3D bioprinting platform, we have developed a fully cellular human *in vitro* model of the proximal tubule interstitial interface comprising renal fibroblasts, endothelial cells, and primary human renal proximal tubule epithelial cells to enable more accurate prediction of tissue-level clinical outcomes. Histological characterization demonstrated formation of extensive microvascular networks supported by endogenous extracellular matrix deposition. The epithelial cells of the 3D proximal tubule tissues demonstrated tight junction formation and expression of renal uptake and efflux transporters; the polarized localization and function of P-gp and SGLT2 were confirmed. Treatment of 3D proximal tubule tissues with the nephrotoxin cisplatin induced loss of tissue viability and epithelial cells in a dose-dependent fashion, and cimetidine rescued these effects, confirming the role of the OCT2 transporter in cisplatin-induced nephrotoxicity. The tissues also demonstrated a fibrotic response to TGFβ as assessed by an increase in gene expression associated with human fibrosis and histological verification of excess extracellular matrix deposition. Together, these results suggest that the bioprinted 3D proximal tubule model can serve as a test bed for the mechanistic assessment of human nephrotoxicity and the development of pathogenic states involving epithelial-interstitial interactions, making them an important adjunct to animal studies.

## Introduction

The kidneys play a central role in the metabolism and elimination of a variety of drugs, with the proximal tubule (PT) being exposed to high concentrations of reactive hydrophilic metabolites at both the luminal surface following filtration of plasma at the glomerulus, as well as the basolateral surface following absorption from the peritubular capillaries. Due to the action of renal xenobiotic transporters expressed in the PT epithelium, pharmaceutical compounds can accumulate and become concentrated in the PT and may then undergo further metabolism by cytochrome P450 enzymes and UDP-glucuronyltransferases (Lohr et al., 1998). While this serves a role in detoxifying these compounds to generate more hydrophilic molecules that are secreted into the urine, highly toxic intermediate metabolites can accumulate and cause damage to the tubular epithelium and surrounding cells (Choudhury and Ahmed, 2006). As such, a major challenge in bringing new drugs to market is the risk of nephrotoxicity, which is often detected late in drug development; attrition due to nephrotoxicity accounts for 2% of preclinical drug attrition but 19% of attrition during more costly phase 3 clinical trials (Redfern, 2010). Post-approval, drug-induced nephrotoxicity accounts for as much as 18–27% of cases of acute kidney injury (AKI) (Loghman-Adham et al., 2012), with up to 36% of these injuries related to commonly used antibiotics such as aminoglycosides (Kleinknecht et al., 1987). While many of these AKI cases are reversible, some drugs can induce chronic renal injury resulting in tubular necrosis, tubulointerstitial inflammation, and fibrosis (Kleinknecht et al., 1987; Choudhury and Ahmed, 2006). Currently, diagnosis of AKI or renal failure relies on elevated creatinine or blood urea nitrogen levels, which do not become reliably clinically significant until the injury is severe (Rahman et al., 2012). The lasting effects of AKI are significant, with 13% of patients requiring continued dialysis and 41% of patients requiring kidney transplant due to renal insufficiency (Vaidya et al., 2008). Better predictive tools for identifying nephrotoxic drugs during the drug development process would therefore reduce the costs associated both with bringing a new drug to market and in treating the downstream effects of AKI, as well as improving patients' lives.

Currently, widely used screening tools for nephrotoxic compounds consist primarily of panels of human and animal renal proximal tubule epithelial cells (RPTEC) or small animal models. However, these systems often fail to accurately predict organ-specific toxicity, either as a result of species-specific differences, or the inability to recapitulate relevant aspects of kidney physiology, including toxicity following xenobiotic transport and biotransformation (Lin and Will, 2012). While freshly isolated primary human RPTEC obviate differences in species specificity, the cells rapidly dedifferentiate and senesce when cultured in isolation, losing expression of key transporters and metabolic enzymes (Hallman et al., 2008; Wieser et al., 2008; Vesey et al., 2009). In the human kidney, the RPTEC exist in close connection with the renal interstitium, defined as the space between the cortical tubules comprising cells, extracellular matrix, proteoglycans, glycoproteins, and interstitial fluid (Lemley and Kriz, 1991). The cell types found in the cortical interstitium include fibroblast-like cells and immune cells, which are interspersed with the microvasculature of peritubular capillaries (Brenner, 2008). These supporting cell types may play a key role in maintaining the continued function of RPTEC, as co-culture of primary RPTEC with endothelial cells results in a robust paracrine signaling network that improves RPTEC proliferation and differentiation (Tasnim and Zink, 2012). Thus, placing primary RPTEC together with supporting interstitial cells in a more native, three-dimensional (3D) architecture may aid in maintaining their function over time, as well as allowing for assessment of additional types of kidney injury that are difficult to model using epithelial cells alone, such as fibrosis (Subramanian et al., 2010).

One of the primary aims of tissue engineering is to use living cells and biomaterials to generate 3D tissues that recapitulate key aspects of the architecture and function of a native tissue or organ. With proper *in vitro* or *in vivo* conditioning, the cells within these structures can respond to soluble and mechanical cues by establishing cell-cell and cell-matrix interactions that mimic some aspects of native tissue (Griffith et al., 2014). It is well established that cells cultured in 3D configurations, such as spheroids or collagen gels, perform differently in functional assays than 2D cultures, and the physiologic responses of cells in 3D more closely approximate responses observed *in vivo* (Godoy et al., 2013). One such means for fabricating these 3D structures is bioprinting. In this approach, bioinks composed of cellular material are extruded in reproducible, geometrically-defined patterns created by the investigator (Ozbolat and Hospodiuk, 2016). The bioink is composed of self-assembling multicellular aggregates that adhere to one another following deposition, leading to formation of complex, patterned tissues (Jakab et al., 2008, 2010). Combining the use of self-assembling multicellular aggregates with computer-controlled bioprinting allows the creation of highly reproducible, scaffold-free tissues that form and mature in the absence of exogenous extracellular matrix that can interfere with direct cell-cell contacts (Norotte et al., 2009). In the current study, Organovo's proprietary bioprinting technology was leveraged to design and create layered tissue models of the human PT that incorporate key interstitial cell types supporting RPTEC to facilitate both cell-cell interactions and paracrine signaling between renal fibroblasts, endothelial cells, and epithelial cells. The resulting engineered tissues were then characterized in three main areas: validation of key physiologic aspects of the native proximal tubule, confirmation of the ability to detect nephrotoxicity using the well-characterized nephrotoxin cisplatin, and demonstration of the utility of the model in evaluating renal fibrosis. The tissues supported physiologically relevant epithelial morphology and function for at least 30 days in culture, and were effectively used to model the role of the organic cation transporter OCT2 in nephrotoxic responses to cisplatin using a combination of biochemical, transcriptional, and histological endpoints. In addition, the tissues demonstrated a fibrotic response to the cytokine TGFβ, a phenotype that is not possible in isolated epithelial cell culture systems. Based on these initial validation studies, this system may be useful in predicting nephrotoxicity of pharmaceutical compounds earlier in the drug development process.

## Materials and methods

### Cell culture

Human umbilical vein endothelial cells (HUVEC) were purchased from BD Biosciences (Franklin Lakes, NJ) and cultured in EGM-2 media with EBM-2 supplements without gentamycin or amphotericin B (Lonza, Basel, Switzerland). Adult renal fibroblasts were purchased from DV Biologics (Yorba Linda, CA) and grown in Fibroblast Cellutions Medium with Fibroblast Cellutions supplement (DV Biologics, Yorba Linda, CA). Primary human RPTEC were purchased from four different commercial vendors (Lonza lot number 0000385391), Sciencell (lot number 11022; Carlsbad, CA), Zen-Bio (lot number RPCT082011; Research Triangle Park, NC), Lifeline Cell Technology (lot number 02685; Frederick, MD) and cultured according to the manufacturer's instructions.

### RPTEC isolation and culture

All kidneys were ethically sourced through the National Disease Research Interchange (Philadelphia, PA). RPTEC cells [available from Samsara Sciences (San Diego, CA)] were isolated using standard methodologies as previously described (Vesey et al., 2009). In brief, upon receipt, kidneys were aseptically unpacked and cleaned to remove any remaining fat pads, ureters, blood vessels or other tissue. Sections of cortical tissue were minced, digested with collagenase, and the collected cells were enriched for epithelium by centrifugation across an iodixanol gradient (Sigma-Aldrich, St. Louis, MO). RPTECs were cultured in GBG™ Epithelial Media (Samsara Sciences, San Diego, CA).

### 3D bioprinting and tissue culture

3D PT ExVive™ Human Kidney Tissue was fabricated as described (Nguyen et al., 2016b). Briefly, cultured renal fibroblasts and HUVEC were combined in a 50:50 ratio and resuspended in thermo-responsive NovoGel® Bio-Ink, and then bioprinted onto 0.4 μm Transwell clear polyester membrane inserts in a 24-well plate (Corning Costar, Corning, NY) using a NovoGen Bioprinter® Instrument (Organovo Inc., San Diego, CA) with previously established protocols (Nguyen et al., 2016a). Following bioprinting, the tissues were cultured in 3D PT Tissue media comprising EGM-2 media with EBM-2 supplements without gentamycin and GBG™ Epithelial Media (Samsara Sciences, San Diego, CA). On culture day 3, primary RPTEC cells were added to the tissues in a suspension of 1.25 × 10^6^ cells/ml in RPTEC media. Tissues were then maintained for up to 30 days in 3D PT Tissue media described above and 2.5% final v/v FBS, with media exchanges every other day. For toxicity and fibrosis studies, tissues were dosed daily with cisplatin and TGFβ in culture media supplemented with a final concentration of 2.5% FBS v/v in both the apical and basolateral compartments beginning at day 14 of culture.

### Metabolic activity and viability assays

Assessment of metabolic activity as a surrogate for tissue viability and health was performed by alamarBlue™ Assay according to the manufacturer's protocol (Thermo Fisher, Carlsbad, CA). Briefly, tissues were washed twice with Dulbecco's phosphate buffered saline (DPBS), and RPTEC media supplemented with 10% v/v alamarBlue reagent was added to each tissue. All tissues were incubated for 2 h at 37°C with 95% relative humidity and 5% CO_2_. After incubation, the alamarBlue solution was removed and fluorescence was measured on a BMG Labtech POLARstar Omega plate reader (Cary, NC) with an excitation filter of 560 nm and an emission filter of 590 nm. Graphed data represent the percent relative fluorescence units (RFU) compared to blank for metabolic activity over time, or the percent RFU compared to vehicle control for toxicity studies.

Lactate dehydrogenase (LDH) activity assay was performed according to the manufacturer's protocol (Abcam, Cambridge, MA). Conditioned media was collected from 3D PT tissues and further diluted in fresh media to ensure that the LDH activity of the sample was within the linear range of the assay. Samples were measured on a microplate reader (BMG Labtech, Cary, NC). LDH activity was determined by standard curve integration of absorbance normalized for volume and duration using GraphPad Prism software (GraphPad, San Diego, CA). Data shown represent the fold change in LDH activity relative to vehicle control for each day of sampling.

### GGT assay

GGT activity was measured according to the manufacturer's protocol (Sigma Aldrich, St. Louis, MO). Tissues were washed twice with DPBS and lysed in GGT assay buffer in a Precellys lysis tube (Precellys, Rockville, MD). Lysate was assessed for GGT activity by comparison to a standard curve integration of absorbance normalized for volume and duration of incubation period at 37°C using GraphPad Prism software (GraphPad, San Diego, CA). Data shown represent the average GGT activity in mIU/ml for analysis of GGT function over time, or percent relative to vehicle for toxicity studies.

### Trans-epithelial electrical resistance (TEER) and permeability measurements

To measure TEER, individual 3D PT tissues cultured for 21 d were removed from the Transwell insert and loaded into an Ussing chamber (Physiologic Instruments, San Diego, CA). Studies were run essentially as previously described (Clarke, 2009). Tissues were bathed in Krebs bicarbonate ringer solution with glucose (115 mM NaCl, 2.4 mM K_2_HPO_4_, 0.4 mM KH_2_PO_4_, 1.2 mM CaCl_2_ dihydrate, 1.2 mM MgCl_2_ hexahydrate, 25 mM ${\text{NaHCO}}_{3}^{-}$, 10 mM glucose; all reagents from Sigma-Aldrich, St. Louis, MO) and buffer was continuously bubbled with carbogen gas (95% O2/5% CO2). After correcting the electrode offset potential and liquid resistance, resistance across the tissues was measured continuously for 1 h.

For apparent permeability (P_app_) measurements, tissues or empty Transwells were washed with DPBS three times and equilibrated to assay buffer (DPBS with 10 mM HEPES pH 7.4) for 10 min at 37°C. Tissues were then dosed with 250 μM Lucifer yellow (Thermo Fisher, Carlsbad, CA) to either the apical or basolateral compartment and fresh assay buffer in the opposing receiver) compartment. Following incubation for 1 h at 37°C, samples were taken from both the apical and basolateral compartments. Fluorescence in each sample was measured on a BMG plate reader with an excitation filter of 490 nm and an emission filter of 540 nm (BMG Labtech, Cary, NC) and normalized to a standard curve for quantification. P_app_ was calculated with equation 1 and 2, where V represents the volume of Lucifer yellow solution, T is the duration of the incubation, D_0_ is the concentration of Lucifer yellow applied to the cells, and A is the growth area of the Transwell insert. Apparent permeability was calculated for each direction (A→B and B→A) and the ratio of these values was then calculated by using Equation (3).

(1) $$P_{appA\to B}=(\frac{V}{A}x\;D_{0}))*(\Delta D/\Delta T)$$

(2) $$P_{appB\to A}=(\frac{V}{A}x\;D_{0}))*(\Delta D/\Delta T)$$

(3) $$\text{Efflux ratio }=\;\frac{\text{PappB}\to \text{A}}{\text{Papp}\;\text{A}\to \text{B}}$$

### ELISA assay for angiotensin-converting enzyme (ACE) and angiotensin II

ACE protein levels in both tissue lysates and conditioned media were detected by ELISA using the manufacturer's instructions (Abcam, Cambridge, MA). Plates were read at 450 nM (BMG Labtech, Cary, NC) within 30 min of addition of the stop solution. Concentrations of the test samples were determined by comparison to the standard curve using GraphPad Prism software (GraphPad, San Diego, CA).

To evaluate ACE enzyme function, 3D PT tissues were treated for 24 h with 5 ng/ml human angiotensin I (Abcam, Cambridge, MA) and angiotensin II was then detected using a competitive ELISA kit from Sigma per the manufacturer's instructions (Sigma-Aldrich, St. Louis, MO). Plates were read at 450 nM within 30 min of addition of the stop solution (BMG Labtech, Cary, NC). The concentration of angiotensin II in the test samples was determined by comparison to the standard curve using GraphPad Prism software (GraphPad, San Diego, CA).

### Histology

3D PT were fixed overnight in 2% paraformaldehyde (Electron Microscopy Sciences, Hatfield, PA). Tissues were oriented for transverse sectioning by pre-embedding in HistoGel (Thermo Fisher, Carlsbad, CA) and were then dehydrated and infiltrated with paraffin by automated processing on a TissueTek VIP tissue processing system (Sakura Finetek USA, Torrance, CA). Tissues were sectioned at 5 μM on a Jung Histocut microtome (Leica Biosystems, Buffalo Grove, IL). Hematoxylin and eosin (H&E) or Gomori's trichrome (TCM) stains were generated using a Leica Autostainer XL (Leica Biosystems, Buffalo Grove, IL) according to manufacturer's instructions. Immunohistochemistry was performed as previously described (King et al., 2013) using the primary antibodies in Table 1. Following overnight incubation with primary antibodies at 4°C, sections were stained with AlexaFluor-conjugated secondary antibodies (Thermo Fisher, Carlsbad, CA) at a 1:200 dilution. For P-gp and SGLT2 detection, tyramide signal amplification was performed according to the manufacturer's instructions (Thermo Fisher, Carlsbad, CA). Slides were counterstained and mounted with FluoroGel II with DAPI (Electron Microscopy Sciences, Hatfield, PA). H&E and TCM images were acquired on a Zeiss Axioskop with Zeiss Zen software (Zeiss Microscopy, Thornwood, NY). Immunofluorescent images were acquired on a Zeiss AxioImager A2 with Zeiss Zen software.

**Table 1:** **List of antibodies used**.

	**Dilution**	**Vendor**
Rabbit α-CD31	1:100	Abcam (Cambridge, MA)
Mouse α-TE7	1:500	EMD Millipore (Temecula, CA)
Mouse α-collagen IV	1:100	Abcam
Rabbit α-E-cadherin	1:50	Abcam
Rabbit α-Pgp	1:500	Abcam
Rabbit α-SGLT2	1:250	Abcam
Rabbit α-Na^+^K^+^ATPase	1:100	Abcam
Mouse α-cytokeratin 18	1:500	Abcam
Rabbit α-PCNA	1:1000	Cell signaling (Danvers, MA)

### Quantification of collagen by Sirius Red/Fast Green staining

Formalin-fixed, paraffin-embedded 3D PT tissues dosed with vehicle or TGFβ were sectioned at 20 μM and dewaxed and rehydrated as described above. Tissues sections were stained using a Sirius Red and Fast Green staining kit and washed extensively according to manufacturer's protocol (Chondrex, Redmond, WA). Following washing, dye was extracted with Dye Extraction Buffer (Chondrex), and measured on a microplate reader (BMG Labtech). Collagenous proteins stained with Sirius Red were detected at 540 nm and non-collagenous proteins stained with Fast Green were detected at 605 nm. The amount of collagen normalized to total protein content was calculated for 4 individual tissue sections from each of 3 tissues. Data shown represents the fold change of normalized collagen content relative to the vehicle control. sRNA Isolation and Quantitative RT-PCR.

### RNA isolation and quantitative RT-PCR

RNA extraction from 3D PT tissues was performed using the Zymo Direct-zol RNA kit according to the manufacturer's instructions (Zymo Research, Irvine, CA). RNA was quantified by spectrophotometry using a NanoDrop 2000 (Thermo Fisher, Carlsbad, CA) and converted to cDNA using SuperScript III First-Strand Synthesis SuperMix according to the manufacturer's instructions (Thermo Fisher, Carlsbad, CA). Amplification reactions were performed with 200 ng of cDNA using TaqMan Gene Expression Array Cards (Thermo Fisher, Carlsbad, CA) with GAPDH amplification as an endogenous housekeeping control gene. TaqMan probe/primer sets are described in Table 2. Amplification was detected on a ViiA7 real-time PCR system (Thermo Fisher, Carlsbad, CA). Duplicate samples from individual tissues were assessed. Relative quantitation (RQ) values for the gene of interest compared to GAPDH were calculated using the formula RQ = (2^−ΔCt^)^*^10000. RQ values for each sample were normalized for KRT18 by dividing the RQ for the gene of interest compared to GAPDH by the RQ for KRT18 compared to GAPDH. The fold change was then calculated by dividing the KRT18-normalized RQ at the experimental day by the KRT18-normalized RQ for day 3 (or day 12 for SGLT2, NHE3, and NAPT2C).

**Table 2:** **Taqman probe/primer sets used**.

**Gene**	**Gene symbol**	**Assay ID**
ACE	*ACE*	Hs00174179_m1
AGT	*AGT*	Hs01586213_m1
Renin	*REN*	Hs00982555_m1
MDR1 (P-gp)	*ABCB1*	Hs00184500_m1
BCRP	*ABCG2*	Hs01053790_m1
AQP1	*AQP1*	Hs01028916_m1
Cubilin	*CUBN*	Hs00153607_m1
Megalin	*LRP2*	Hs00189742_m1
OCT2	*SLC22A2*	Hs01010723_m1
OAT1	*SLC22A6*	Hs00537914_m1
OAT3	*SLC22A8*	Hs00188599_m1
MATE1	*SLC47A1*	Hs00217320_m1
MATE2K	*SLC47A2*	Hs00945650_m1
SGLT2	*SLC5A2*	Hs00894642_m1
NAPT2C	*SLC34A3*	Hs02341449_m1
NHE3	*SLC9A3*	Hs00903842_m1
COL1A1	*COL1A1*	Hs00164004_m1
CTGF	*CTGF*	Hs01026927_g1
PDGFRB	*PDGFRB*	Hs01019589_m1
FAP	*FAP*	Hs00990806_m1

### LC-MS/MS-based detection of renal transporters

The ProteoExtract® Native Membrane Protein Extraction Kit was purchased from Calbiochem/MerckMillipore (Darmstadt, Germany). The protein quantification bicinchoninic acid (BCA) assay kit, sequencing-grade trypsin, iodoacetamide (IAA), and dithiothreitol (DTT) were purchased from Pierce Biotechnology (Rockford, IL). Chloroform, high-performance liquid chromatography (HPLC)-grade acetonitrile/methanol, and formic acid were purchased from Fischer Scientific (Fair Lawn, NJ). Ammonium bicarbonate (98% purity) and sodium deoxycholate (DOC, 98% purity) were obtained from Thermo Fisher Scientific (Rockford, IL) and MP Biomedicals (Santa Ana, CA), respectively.

3D PT tissues cultured for 21 days or pieces of normal human kidney cortex were subjected to targeted proteomic analysis for OAT1, OAT3, OCT2, and P-gp and human serum albumin (HSA, internal standard). Total membrane was isolated from the 3D PT tissues and normal human kidney cortex using a protocol previously described (Prasad et al., 2014). Quantification of the total protein concentration was determined using a BCA assay and the final membrane fraction was diluted to a working concentration of 0.5 μg membrane protein/μL. Total membrane proteins were spiked with 20 uL of HSA (10 ug/mL) and subsequently reduced, denatured, alkylated and digested using a previously reported protocol (Wang et al., 2015). All samples were digested and the surrogate peptides generated by trypsin digestion were monitored by LC-MS/MS as described below. Peptides unique for each transporter were selected based on *in silico* selection criteria (Kamiie et al., 2008; Prasad and Unadkat, 2014). Surrogate peptides were monitored using an API 3000 triple quadrupole tandem mass spectrometer (Applied Biosystems/MDS Sciex, Foster City, CA) coupled to an Agilent® 1290 Infiniti II™ UPLC system (Agilent, Palo Alto, CA).

Briefly, a UPLC column (Acquity UPLC® HSS T3 1.8 μm, 2.1 × 100 mm, Waters), with a Security Guard column (C18, 4 × 2.0 mm) from Phenomenex (Torrance, CA), was eluted (0.3 mL/min) with a gradient mobile phase consisting of water and acetonitrile (with 0.1% formic acid). Peptides were eluted with a gradient of 3–60% acetonitrile over 23 min, followed by a return to starting conditions for 4 min to re-equilibrate the system. The injection volume was 5 μL (~2.5 μg of total protein). The mass spectrometer was set up to run a multiplexed MRM experiment for the optimal peptides. The MRM transitions for the analyte peptides were monitored using LC-MS/MS parameters in ESI positive ionization mode. The total peak area of the selected peptide was normalized to HSA from the same sample and were determined using Skyline (MacCoss Lab, University of Washington).

### Glucose uptake colorimetric assay

Glucose uptake in 3D PT tissues was detected and quantified according to the manufacturer's protocol (Abcam, Cambridge, MA). Insulin-Transferrin-Selenium (Gibco, Carlsbad, CA) was used to stimulate glucose uptake and canagliflozin (Santa Cruz Biotech, Dallas, TX) was used to inhibit SGLT2 function. Tissues were starved overnight in DPBS/HEPES, pH 7.4 prior to assay. Tissues were then pretreated with 1X insulin or 500 μM canagliflozin for 20 min, followed by addition of 1 mM 2-deoxyglucose. Tissues were washed extensively with PBS and lysed in extraction buffer in Precellys lysis tubes (Precellys, Rockville, MD). 2-deoxyglucose uptake was measured at OD412 nm on a microplate reader (BMG Labtech, Cary, NC) and results were graphed as fold change relative to control using GraphPad Prism Software (GraphPad, San Diego).

### Vectorial transport of rhodamine 123

3D PT tissues were washed with DPBS three times and equilibrated to assay buffer (DPBS supplemented with 10 mM HEPES, pH 7.4) for 10 min at 37°C. Both apical and basolateral sides of tissues were then pre-incubated for 20 min at 37°C in assay buffer in the presence or absence of 5 μM zosuquidar (Sigma Aldrich, St. Louis, MO). Following pre-treatment, tissues were dosed on the basolateral side with 1 μM rhodamine 123 (Molecular Probes, Eugene, OR) with or without 5 μM zosuquidar for 2h at 37°C. After incubation, the tissues were washed with cold assay buffer, fixed with 2% PFA, and cryosectioned. Images were captured at the same exposure time across all conditions. Fluorescence intensity, corrected for background and relative area, was calculated in Image J (National Institutes of Health, Bethesda, MD) and graphed as fold change relative to control using GraphPad Prism Software (GraphPad, San Diego).

### Statistical analysis

Statistics were calculated using GraphPad Prism software (La Jolla, CA). Data shown is the mean ± SEM. Statistical significance (*P* < 0.05) was calculated by *t*-test with Dunnet's post-test, one-way ANOVA, or two-way ANOVA as appropriate.

## Results

### Development and characterization of a 3D model of the tubulointerstitial interface of the human PT

**Figure 1:**
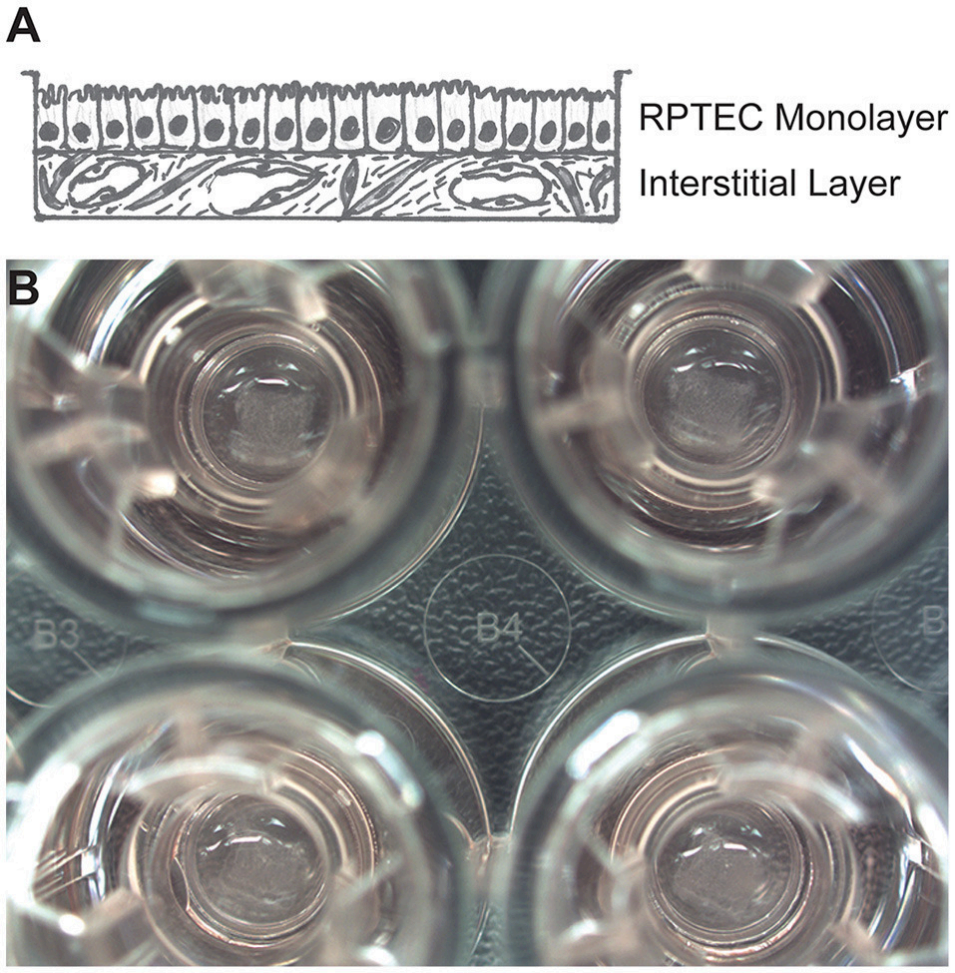
**Description of a 3D model of the PT tubulointerstitial interface printed with the NovoGen Bioprinter® instrument. (A)** Schematic diagram showing a multicellular interstitial layer underlying a basement membrane that supports an epithelial monolayer. **(B)** Macroscopic view of 3D PT tissues positioned on Transwell inserts in a standard 24-well plate (Corning Costar, Corning, NY).

Cultured primary human RPTECs have a finite lifespan in culture before undergoing epithelial-to-mesenchymal transition or senescence, with accompanying loss of morphology and function (Wieser et al., 2008). Abundant evidence supports the notion that an appropriate microenvironment, including 3D architecture and supporting cell types, can help maintain and support the continued health and function of polarized epithelia (Kunz-Schughart et al., 2006; Bryant and Mostov, 2008; Nagle et al., 2011; Li et al., 2014). To develop a 3D human system for studying nephrotoxicity, the Organovo NovoGen Bioprinter system was used to create a model of the PT tubulointerstitial interface (ExVive tissues). As shown in the schematic of Figure 1A, tissues were designed with a basal multicellular interstitial layer composed of primary human renal fibroblasts and HUVEC, and an apical monolayer of polarized primary human RPTEC supported by a basement membrane. Use of the bioprinter allowed reproducible generation of spatially-defined tissues created on standard multi-well Transwell inserts (Figure 1B, King et al., 2016).

**Figure 2:**
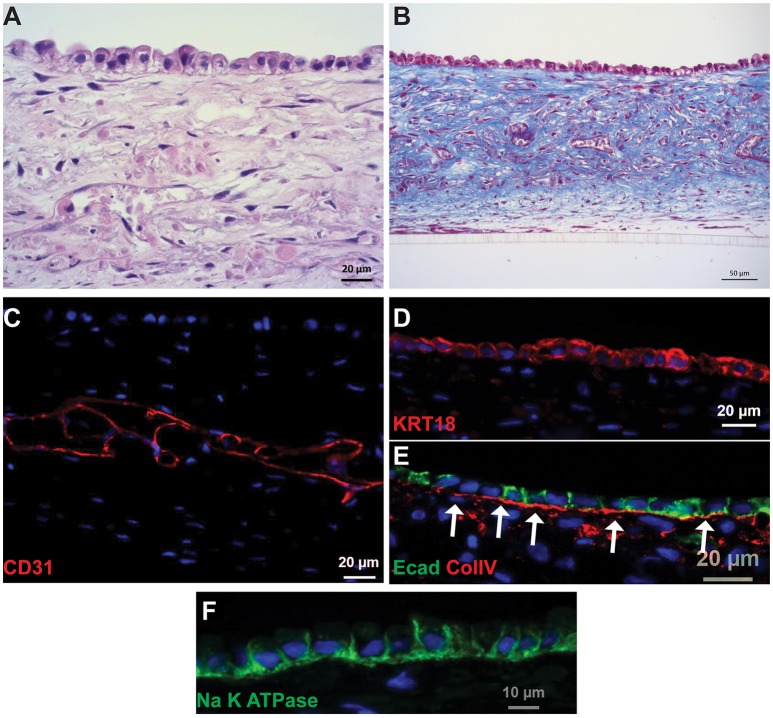
**Histological characterization of 3D PT tissues**. Representative images of tissues cultured for 14 days are shown. **(A)** H&E stain showing fully cellular tissue and organization of interstitial and epithelial layers. **(B)** Gomori's trichrome stain showing deposition of collagen throughout the tissue. **(C)** The interstitial layer demonstrates extensive endothelial cell-lined networks (red, CD31). **(D)** RPTEC form a monolayer and express cytokeratin 18 (red). **(E)** A collagen IV-rich basement membrane underlies the epithelial cells and E-cadherin localizes to tight junctions between the cells (red, collagen IV; green, E-cadherin). **(F)** Na+K+ATPase localizes to the basolateral membrane of RPTEC.

Following culture for 14 days, the PT tissues were analyzed for tissue organization, cell morphology, and retention of endothelial and epithelial markers (Figure 2, King et al., 2016). A hematoxylin and eosin (H&E) stain of the 3D tissues showed an interstitial layer with low cell density composed of spindle-shaped fibroblasts and areas of HUVEC undergoing remodeling to form endothelial cell-lined networks (Figure 2A). A monolayer of RPTEC cells was observed immediately above the interstitium, with columnar morphology and basally oriented nuclei. The interstitial cells themselves secreted abundant ECM as shown by Gomori's trichrome stain, with fibrillar structures visible surrounding the endothelial cell networks in the middle of the tissue as well as underlying the epithelial layer (Figure 2B). The putative endothelial cell networks observed by H&E and trichrome expressed CD31 and demonstrated that the HUVEC had organized to form open spaces lined by endothelial cells (Figure 2C). Separating the interstitium from the epithelium was a collagen IV-rich basement membrane immediately adjacent to the basal side of the epithelial cells (Figure 2E). The RPTEC cells in the 3D PT model expressed cytokeratin 18 uniformly across the monolayer (Figure 2D) with E-cadherin localized laterally between adjacent cells (Figure 2E). Polarized distribution of Na^+^/K^+^ ATPase to the basolateral membrane of RPTEC was also observed (Figure 2F).

As a first measure of longevity, tissues were assessed for their ability to reduce resazurin in an alamarBlue assay or to exhibit epithelial-specific GGT activity over 4 weeks in culture. The total tissue metabolic activity of the 3D PT tissues increased between day 10 and day 15 and then reached a plateau that was maintained out to day 30, with low variability from tissue to tissue as demonstrated by the coefficient of variation (% CV; Supplemental Figure 1A). The increase could reflect an increase in metabolic activity or an increase in the overall size of the tissue. The 3D PT tissues exhibited an increase in GGT activity from 5 mIU/ml at day 7 to 30 mIU/ml at day 30, while interstitium controls showed negligible activity (Supplemental Figure 1B, King et al., 2016). As seen in the metabolic activity assay, analysis of the % CV in the GGT assay suggested low tissue-to-tissue variability. Because of an inability to normalize this readout to total protein content, it is unclear whether the increase reflects an increase in enzymatic activity, epithelial cell number or overall tissue size. While the exact mechanisms are not well understood, the lack of decline in viability and GGT over time suggests that there is sustained tissue health over the 4 week timecourse. Together, these findings demonstrate the formation of a robust 3D model of the renal tubulointerstitial interface capable of supporting RPTEC morphology, viability and function for at least 4 weeks.

### Characterization of barrier function

To measure the barrier function of the 3D PT tissues, trans-epithelial electrical resistance (TEER) measurements were performed using an Ussing chamber after 21 days in culture. Table 3 and King et al. (2016) shows the average area-corrected resistance values for 3D PT tissues, which averaged 18.1 Ω ^*^cm^2^. The apparent permeability (P_app_) was also measured in 3D PT tissues by addition of Lucifer yellow to the apical (A) or basolateral (B) compartment of the Transwell and detection of the fluorophore in the opposite compartment as a function of time. 3D PT tissues exhibited an average A→B P_app_ value of 6.31 × 10^−6^ ± 0.83 × 10 cm/s^−6^ and a B→A P_app_ value of 5.33 × 10^−6^ ± 1.06 × 10^−6^ cm/s, for an efflux ratio of 0.84 (Table 3). As the B→A P_app_/A→B P_app_ efflux ratio approached unity, the data shows active transport played a minimal role in the disposition of Lucifer yellow in the model. Empty Transwells devoid of tissues exhibited much higher permeability, with an average A→B P_app_ value of 308 × 10^−6^ ± 3.6 × 10^−6^ cm/s, supporting the conclusion that the 3D PT tissues themselves were responsible for the observed barrier function. When compared to literature findings, these results demonstrate that the barrier formed by the RPTEC cells in the 3D PT tissues is leakier than isolated epithelial monolayers but more characteristic of the barrier observed for the PT *in vivo* (Boulpaep and Seely, 1971; Liang et al., 1999).

**Table 3:** **Measurement of TEER and apparent permeability in 3D PT tissues**.

**Tissue**	R(Avg) Ω^*^cm^2^	**P_app_(A→B) cm/s**	**P_app_ (B→A) cm/s**	**Efflux ratio**
3D PT	18.1 ± 1.3	6.31 × 10^−6^ ± 0.83 × 10^−6^	5.33 × 10^−6^ ± 1.06 × 10^−6^	0.84
Empty well	ND	308 × 10^−6^ ± 3.63 × 10^−6^	ND	N/A

*Tissues were cultured for 14–21 days, and TEER was measured using an Ussing chamber. Apparent permeability was measured by directional detection of Lucifer yellow and calculation of the efflux ratio of B→A:A→B. Data shown is the mean of 6 replicates plus or minus the standard error of the mean. ND, not detected; N/A, not applicable*.

### Assessment of the intrarenal renin-angiotensin system (RAS)

**Figure 3:**
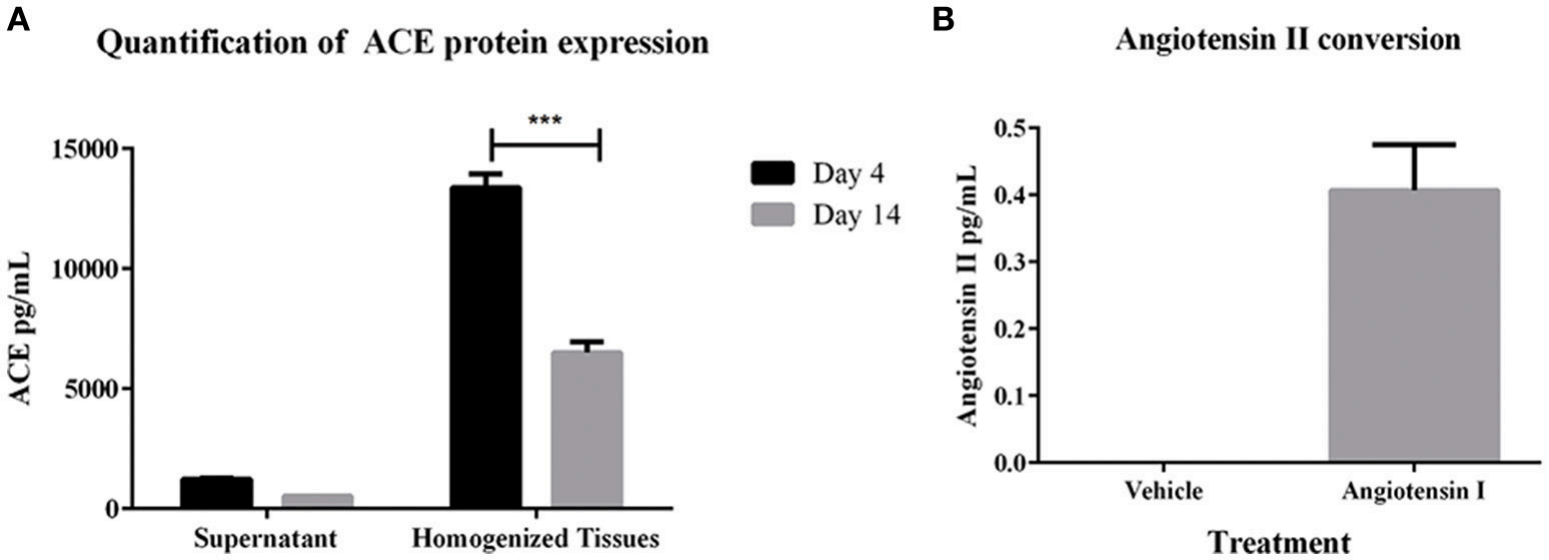
**RAS pathway component activity in 3D PT tissues. (A)** Expression levels of ACE in supernatant and lysates from 3D PT tissues cultured 4 or 14 days. **(B)** Detection of angiotensin II following ACE-mediated conversion of exogenous angiotensin I. Data shown is the mean of duplicate measurements from 3 independent tissue samples plus or minus the standard error of the mean. ^***^p < 0.001.

To determine whether the 3D PT tissues retained a viable RAS, gene expression of several members of the pathway were first measured. Gene expression analysis of the tissues over 30 days in culture showed detectable levels of ACE, angiotensinogen (AGT), angiotensin receptor I (AGTR1), and renin (Supplemental Table 1). Consistent with the gene expression data, ACE protein was detected in both conditioned media and tissue lysates, with higher detection in the tissue lysates (Figure 3A). This may correlate with the observed expression of ACE in the brush border of the PT (Kobori et al., 2007). To evaluate the function of ACE, 3D PT tissues were dosed with 5 ng/ml human angiotensin I for 24 h and assessed for the ability to convert angiotensin I to angiotensin II. Following stimulation, angiotensin II was detected in 3D PT tissues at 0.4 pg/ml (Figure 3B). Thus, the 3D PT tissues exhibited physiologically relevant features of the *in vivo* PT, including development of barrier functions and conversion of angiotensin I to angiotensin II.

### Analysis of renal transporters in 3D PT tissues

**Figure 4:**
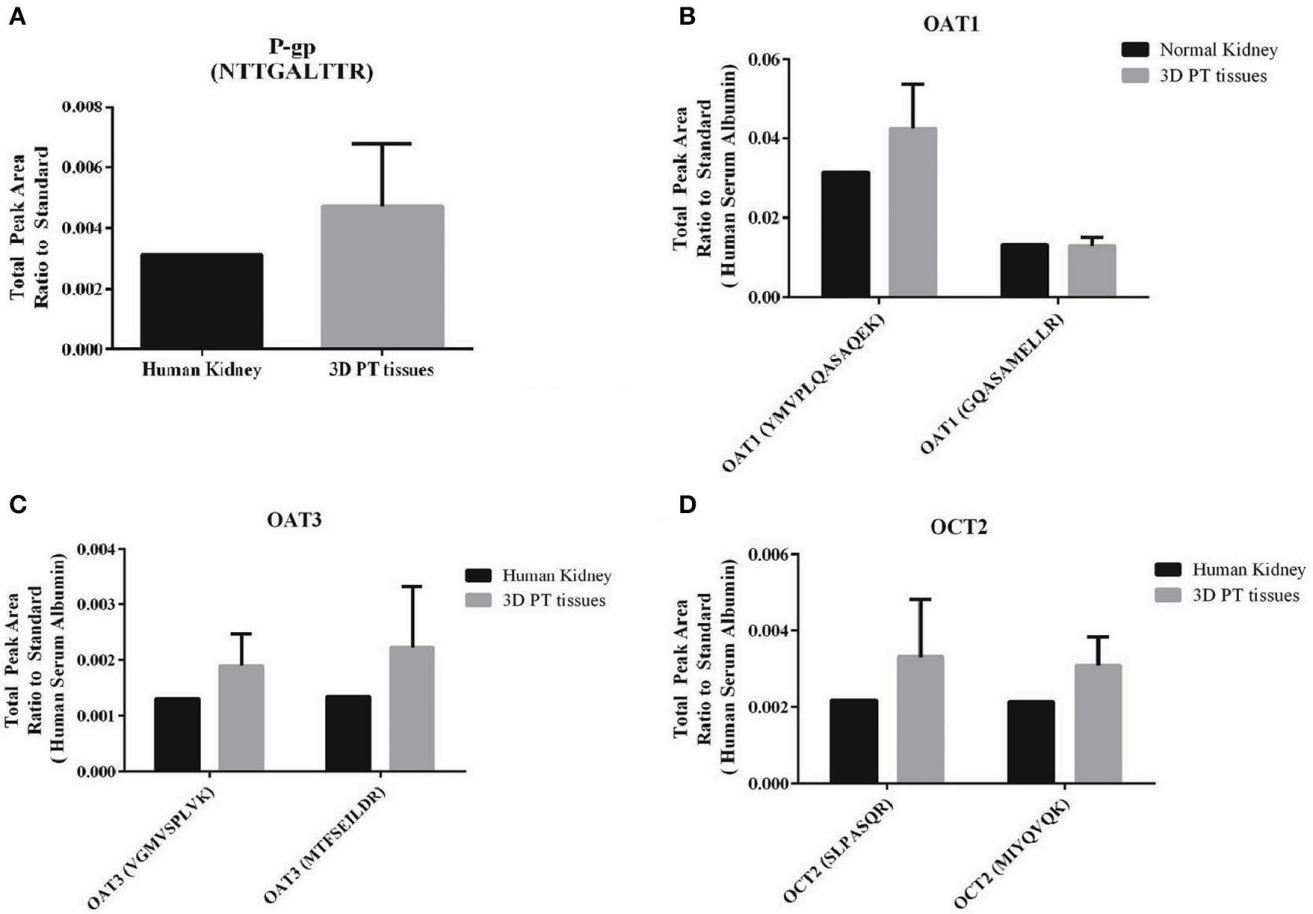
**Detection of renal transporter peptides by LC-MS/MS**. After 14 days in culture, 3D PT tissues or human kidney cortical tissue were subjected to tryptic digestion and relative quantitation of renal transporter peptides by mass spectrometry. **(A)** Detection of P-gp. **(B)** Detection of OAT1 using 2 different peptides. **(C)** Detection of OAT3 using 2 different peptides. **(D)** Detection of OCT2 using 2 different peptides. Data shown is the mean from 5 independent tissue samples plus or minus the standard error of the mean.

A key feature of the PT that relates to its susceptibility to nephrotoxicity is the expression and function of renal transporters, which take up or efflux compounds from the capillaries surrounding the PT or the glomerular filtrate in the lumen of the tubule. Primary human RPTEC dedifferentiate rapidly when cultured in 2D, exhibiting varying levels of renal transporters and a range of cellular morphologies depending on the time and method of culture (Supplemental Figure 2, Wieser et al., 2008; Vesey et al., 2009). We hypothesized that culturing low-passage primary human RPTEC on a relevant renal interstitium would preserve transporter expression and function. To validate the use of the 3D PT model for transporter-dependent toxicity studies, tissues were first analyzed for relative expression levels of key renal transporter genes by qPCR (Supplemental Table 1). The 3D PT tissues exhibited stable levels of expression of many important renal transporters, as well as the epithelial ion channel NHE3 and the sodium phosphate transporter NAPT2C. As an additional assessment of the expression of key renal transporters at the protein level, 3D PT tissues were evaluated by mass spectrometry for detection of peptides corresponding to P-gp, OAT1, OAT3, and OCT2 (Figure 4). The expression of renal transporter proteins was compared to that of human cortical kidney tissue. For each transporter evaluated, the 3D PT tissues exhibited low variation in levels of expression across 5 independent tissues from 2 separate experiments. For OAT1, OAT3, and OCT2, detection was evaluated using 2 different peptides, which showed slight differences in the total peak area ratio of the protein detected (Figures 4B–D) attributable to the variation in ionization efficiency of the two different peptides analyzed by LC-MS/MS. For each transporter evaluated, the 3D PT tissues exhibited peak area values comparable to that observed for human kidney cortex tissue. Thus, the 3D PT tissues exhibit physiologically-relevant levels of renal transporter proteins, enabling continued evaluation of transporter function in this system.

**Figure 5:**
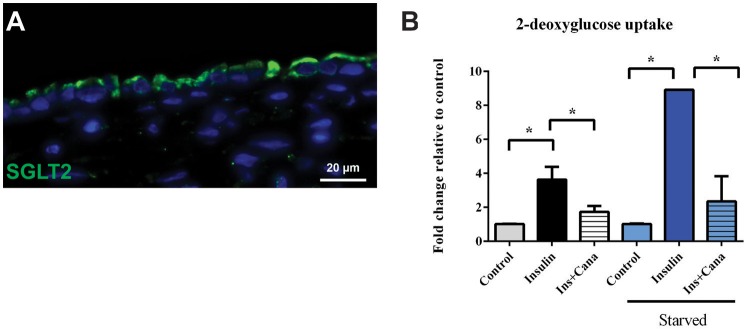
**SGLT2 transporter localization and function. (A)** After 14 days in culture, 3D PT tissues were stained with antibodies against SGLT2 (green). **(B)** Tissues were assessed for retention of the non-metabolizable glucose analog 2-DG in a colorimetric assay in the presence or absence of the glucose uptake inducer insulin or the SGLT2 inhibitor canalgliflozin (Cana). Starved tissues are indicated by the blue family of bars. Data shown is the mean of triplicate measurements across 6 independent tissue samples plus or minus the standard error of the mean. ^*^p < 0.01 between the groups compared by one-way ANOVA.

**Figure 6:**
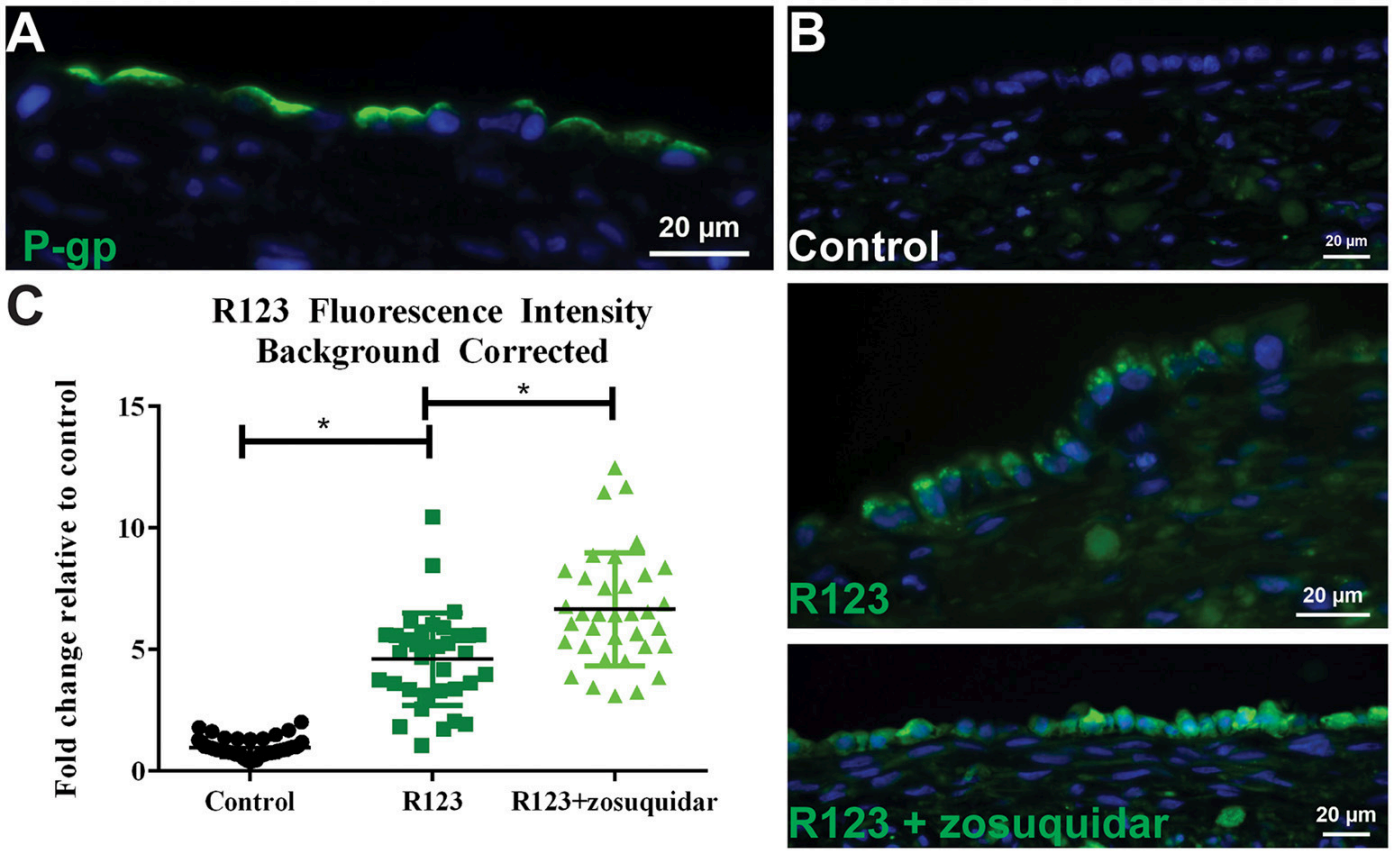
**P-gp transporter localization and function. (A)** After 14 days in culture, 3D PT tissues were stained with antibodies against P-gp (green). **(B)** Tissues were exposed to 5 μM zosuquidar alone, 10 μM rhodamine 123, or rhodamine 123 + zosuquidar for 2 h. Tissues were snap fixed, cryosectioned, and all tissues were imaged at the same exposure time. **(C)** Fluorescence intensity was quantified in Image J. Data shown represents the mean of duplicate measurements from at least 6 independent tissue samples plus or minus the standard error of the mean. ^*^p < 0.0001 between the groups as compared by one-way ANOVA.

To assess both uptake and efflux transporter function in the 3D PT model, the glucose uptake transporter SGLT2 and the xenobiotic efflux transporter P-gp were selected for functional analysis (Figures 5, 6 and King et al., 2016). As shown in Figure 5A, SGLT2 protein expression was detected primarily at the apical surface of RPTEC on 3D PT tissues. This pattern matches what is seen *in vivo* in the human PT (Brenner, 2008). To evaluate SGLT2 transporter function, tissues were kept in either normal tissue maintenance media or starved of glucose for 24 h, followed by stimulation of glucose uptake by insulin in the presence or absence of the SGLT2 transport inhibitor canagliflozin (Figure 5B). In tissues maintained in normal tissue media, treatment with insulin induced a 4-fold increase in intracellular 2-DG, which decreased by 50% upon co-administration of the SGLT2 inhibitor canagliflozin (Figure 5B, black and gray bars). This suggests that there is functional SGLT2 transport in the tissues, and that other transport mechanisms are also contributing to global glucose uptake. When tissues were starved overnight, insulin induced an 8-fold increase in glucose uptake, which was significantly reduced by canagliflozin to levels indistinguishable from the control tissues (Figure 5B, blue bars). As expected, starvation increased glucose uptake by 3D PT tissues beyond that observed for tissues cultured in normal media as the tissues sought to re-establish glucose homeostasis lost during culture in the absence of glucose, and SGLT2 appears to play a role in this process.

To assess P-gp mediated efflux capabilities in 3D PT tissues, we first wanted to determine the localization of the transporter protein. As expected for native proximal tubule, P-gp protein expression was detected at the apical surface of the RPTEC cells in the 3D PT model (Figure 6A). To evaluate P-gp function, 3D PT tissues were loaded with rhodamine 123 (R123) in the presence or absence of zosuquidar, a P-gp inhibitor. Following uptake, tissues were washed and cryosectioned to detect the presence of R123 in the RPTEC of the PT model (green). Tissues treated with buffer alone exhibited no green fluorescence (control), while tissues treated with R123 exhibited punctate fluorescent expression in the cytoplasm of the RPTEC. Upon blocking P-gp-mediated efflux with zosuquidar, an increase in accumulated fluorescence was observed in the epithelium with the RPTEC monolayer fluorescing uniformly throughout the cytoplasm (Figure 6B). Image quantification showed that tissues exposed to R123 exhibited a 4-fold increase over control tissues, while treatment with R123 plus zosuquidar resulted in a 6-fold increase in fluorescence over control tissues and a significant increase compared to R123- treatment alone (Figure 6C). Thus the 3D PT tissues exhibited stable expression of renal transporters over time, and functional activity of the endogenous substrate transporter SGLT2 and the xenobiotic transporter P-gp were verified.

### Assessment of cisplatin nephrotoxicity using 3D PT tissues

**Figure 7:**
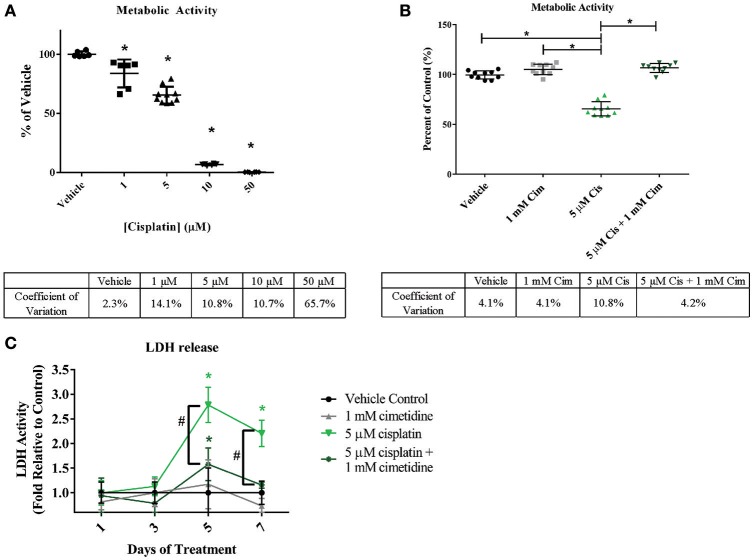
**Reduction of overall viability in 3D PT tissues in response to cisplatin and rescue by the OCT2 inhibitor cimetidine**. Tissues were treated daily for 7 days with increasing doses of cisplatin **(A)** or with 5 μM cisplatin (Cis) in the presence or absence of 1mM cimetidine (Cim; **B**). Cimetidine alone was also tested as a control **(B)**. Overall tissue viability was measured by alamarBlue metabolism. Data shown is indicative of duplicate measurements from 3 individual tissues. Asterisks (^*^) indicate *p* < 0.0005 compared to vehicle control by one-way ANOVA and Dunnett's post-test. % CV across multiple 3D PT tissues from separate experiments at each time point is shown below each graph. **(C)** Daily LDH release from tissues treated with cimetidine, cisplatin, or a combination of cisplatin and cimetidine as a measure of toxicity. Data shown represents the mean of duplicate measurements from 3 independent tissue samples plus or minus standard deviation. Asterisks (^*^) indicate *p* < 0.002 between vehicle and treatment groups as assessed by two-way ANOVA. Number sign (#) indicates *p* < 0.001 between 5 μM cisplatin and 5 μM cisplatin + 1 mM cimetidine as assessed by two-way ANOVA.

Cisplatin is a chemotherapeutic agent with multiple mechanisms of action that lead to nephrotoxicity, including generation of reactive oxygen species and formation of toxic glutathione conjugates following concentration of the molecule in RPTEC by renal uptake transporters including OCT2 (Hanigan and Devarajan, 2003; Yonezawa et al., 2005). In addition, cisplatin has been reported to lead to tubulointerstitial fibrosis (Guinee et al., 1993). To assess whether the 3D PT tissues could manifest OCT2-dependent cisplatin toxicity, tissues were exposed daily to cisplatin in the presence or absence of the OCT2 inhibitor cimetidine followed by measurement of overall viability, release of LDH, and histological analysis (Figure 7 and King et al., 2016). Tissues treated with cisplatin exhibited a significant decrease in alamarBlue metabolism at doses as low as 1 μM, with an LD 50 value of 5.72 μM and complete loss of viability at 10 μM (Figure 7A). No loss of viability was observed in tissues treated with cimetidine alone compared to the vehicle control (Figure 7B). While tissues treated with 5 μM cisplatin exhibited a nearly 50% decrease in viability, tissues treated with a combination of cisplatin and cimetidine exhibited viability levels indistinguishable from vehicle or cimetidine-only controls (Figure 7B). LDH release, indicative of toxicity, peaked at treatment day 5 in tissues treated with 5 μM cisplatin alone, with an observed 3-fold increase over vehicle controls (Figure 7C). Tissues treated with cisplatin plus cimetidine did not exhibit the same damage response, showing only slightly elevated levels of LDH release compared to vehicle at day 5 and indistinguishable levels vs. control-treated tissues by day 7 (Figure 7C).

**Figure 8:**
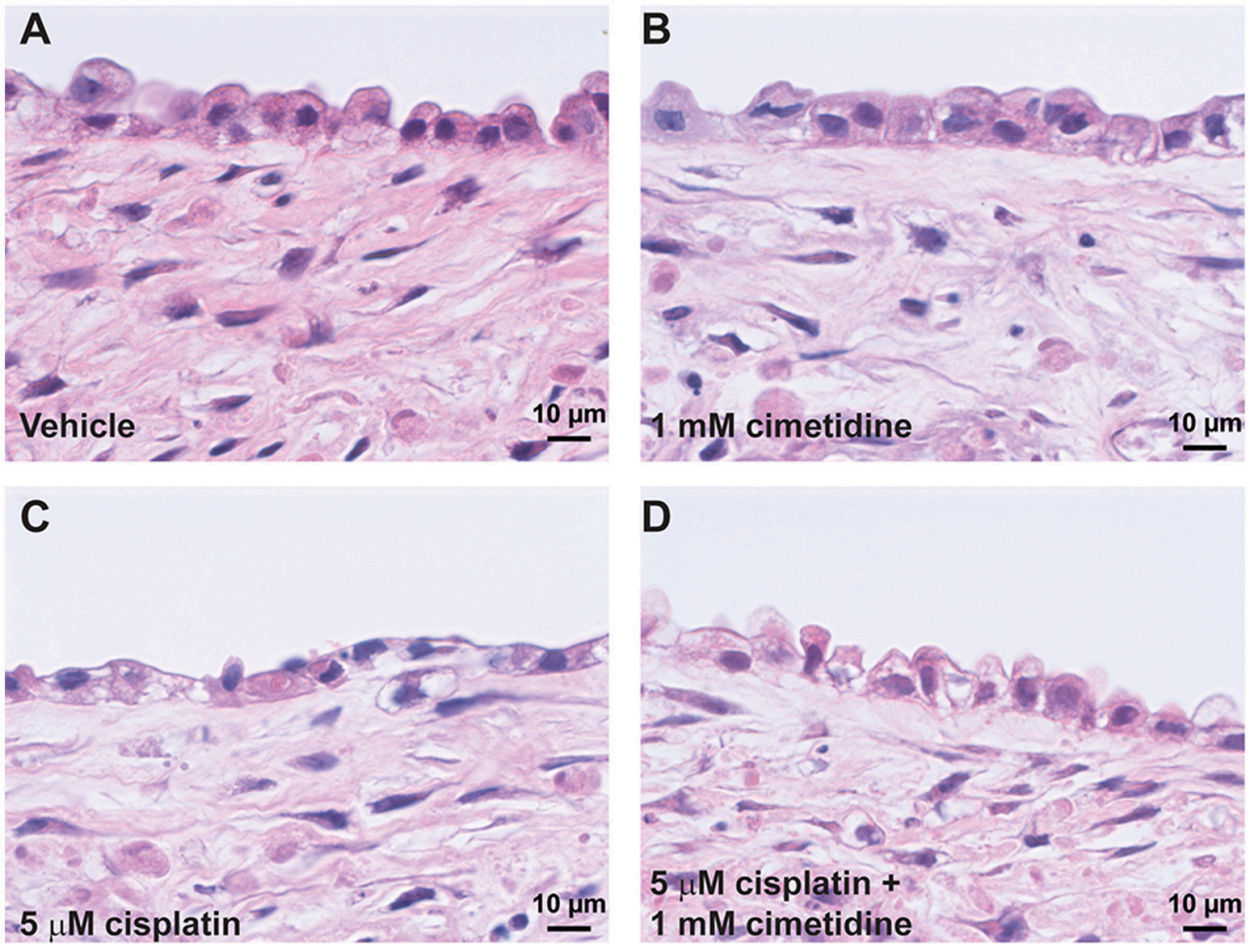
**Histological analysis of cisplatin toxicity**. Representative H&E images are shown for tissues dosed daily for 7 days with vehicle **(A)**, 1 mM cimetidine **(B)**, 5 μM cisplatin **(C)**, or 5 μM cisplatin + 1 mM cimetidine **(D)**.

**Figure 9:**
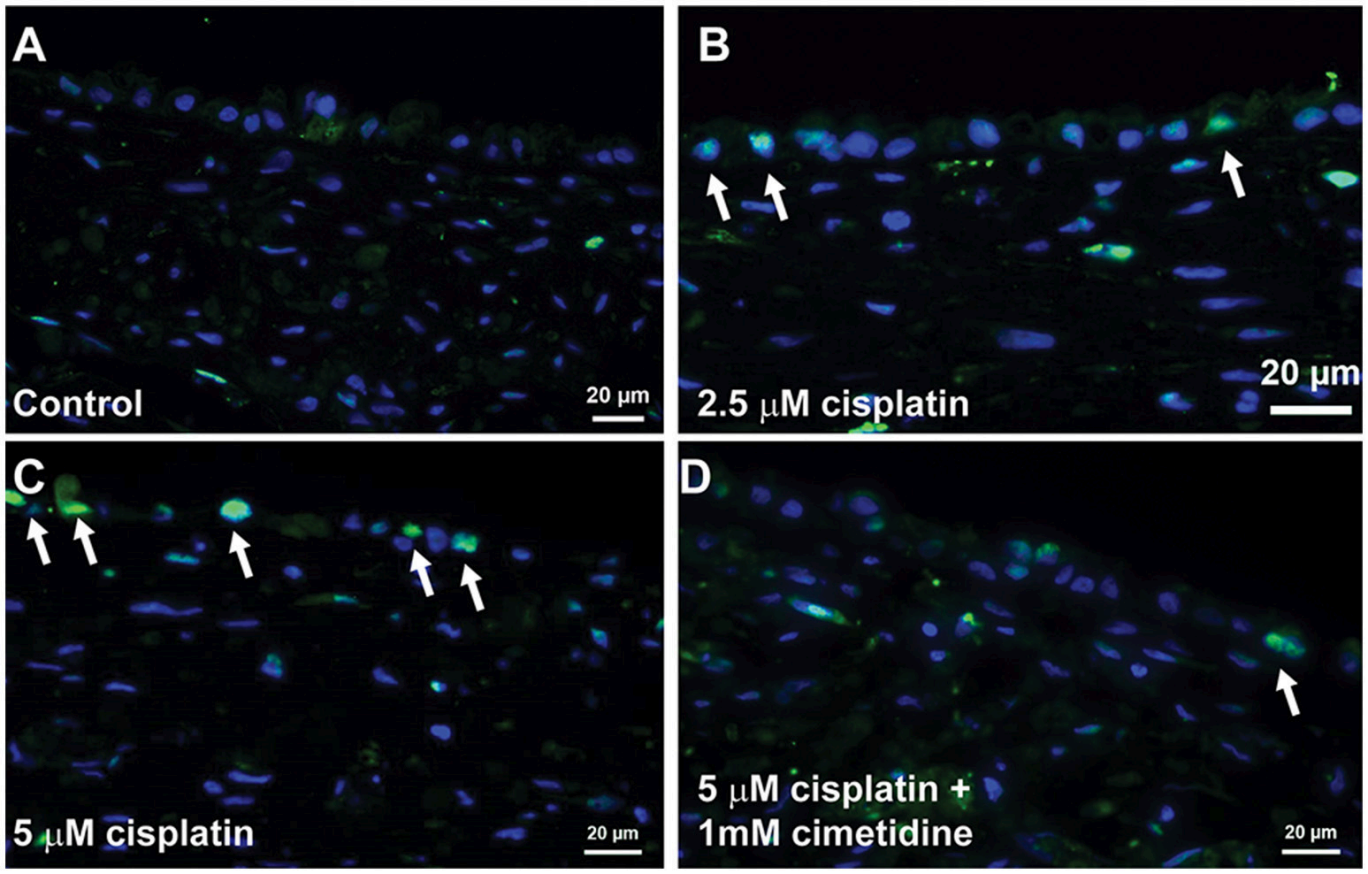
**Proliferation of RPTEC in response to damage**. Tissues were dosed daily for 7 days with vehicle **(A)**, 2.5 μM cisplatin **(B)**, 5 μM cisplatin **(C)**, or 5 μM cisplatin + 1 mM cimetidine **(D)** and stained with an antibody against PCNA. Proliferating cells are marked with white arrows.

Histological analysis by H&E staining confirmed the loss of epithelial viability in response to cisplatin (Figure 8, King et al., 2016). Vehicle or cimetidine-only tissues exhibited healthy, columnar RPTEC with round nuclei (Figures 8A,B), while tissues treated with 5 μM cisplatin exhibited a more squamous morphology and loss of nuclei (Figure 8C). Tissues treated with cisplatin plus cimetidine exhibited a substantial improvement in epithelial morphology vs. cisplatin alone, with partial restoration of nuclear localization and columnar RPTEC (Figure 8D). The loss of epithelial cells in response to cisplatin and rescue by cimetidine was further supported by changes in GGT activity in treated tissues, though changes in enzymatic activity or overall tissue mass cannot be fully excluded (Supplemental Figure 3A, King et al., 2016). To evaluate RPTEC proliferation in response to damage induced by cisplatin, tissues were stained for proliferating cell nuclear antigen (PCNA). Vehicle or cimetidine-treated control tissues exhibited low levels of RPTEC proliferation; however, a dose-dependent proliferative response was observed in tissues treated with cisplatin (Figure 9). This increased proliferation in the RPTEC of 3D PT tissues was decreased by co-administration of cimetidine. Thus, the 3D PT tissues were able to recapitulate nephrotoxicity after exposure to clinically-relevant doses of cisplatin and confirm the role of the OCT2 transporter as a mechanism of nephrotoxicity induction.

### Demonstration of TGFβ-induced fibrotic effects on the interstitium of 3D PT tissues

**Figure 10:**
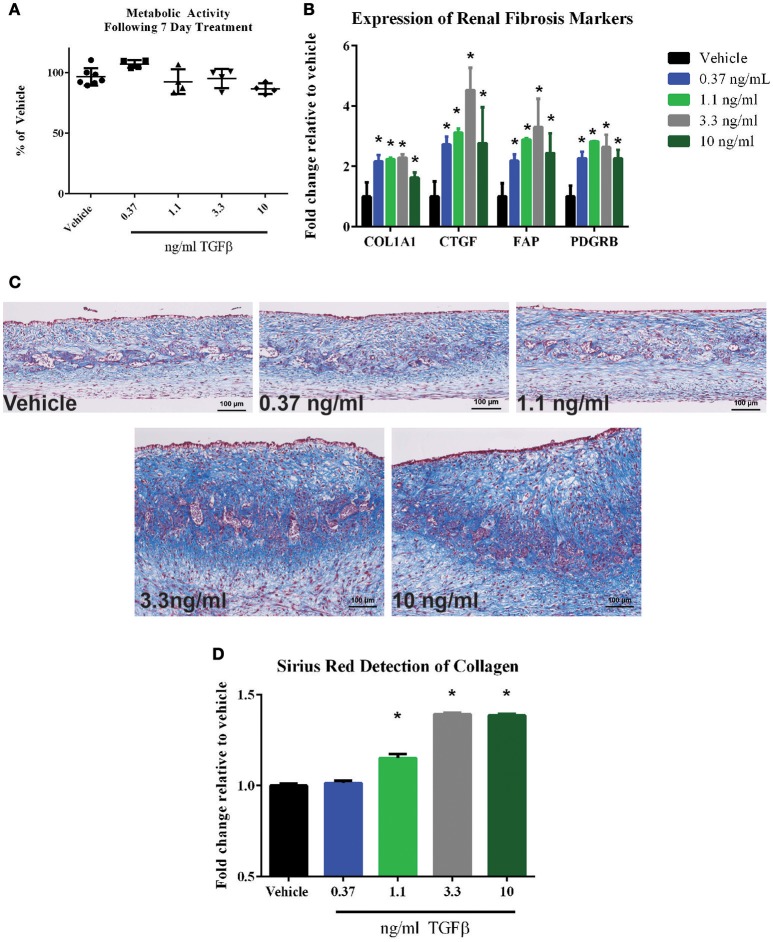
**Response of 3D PT tissues to TGFβ**. Tissues were dosed daily for 7 days with vehicle, 0.37 ng/ml TGFβ, 1.1 ng/ml TGFβ, 3.3 ng/ml TGFβ, or 10 ng/ml TGFβ. **(A)** alamarBlue analysis of overall tissue metabolic activity. Data shown is the average of 3 tissue samples per condition and is represented as the fold change relative to the vehicle control. No statistically significant differences were detected between treatment groups. **(B)** Induction of the fibrotic markers collagen I (COL1A1), connective tissue growth factor (CTGF), fibroblast-activating protein (FAP), or platelet-derived growth factor receptor β (PDGFRB) was assessed by quantitative RT-PCR. Data shown is the average of 3 tissue samples per condition and is represented as the fold change relative to the vehicle control. ^*^p < 0.0001 for each condition compared to vehicle. **(C)** Representative Gomori's trichrome stains for ECM deposition are shown. **(D)** Quantification of Sirius red-stained collagen in tissue sections is shown. Data represents the average of 4 technical replicates per tissue, 3 tissues per condition and is represented as the fold change relative to the vehicle control following normalization to total protein content as measured by Fast Green staining. ^*^p < 0.05 for each condition compared to vehicle.

One key deficit of standard 2D epithelial models is their inability to detect effects on the renal interstitium, which in turn could go on to impact global tissue structure and function. To demonstrate the ability of the 3D PT tissues to respond to agents that impact cells of the interstitial compartment, the ability of TGFβ to induce a fibrotic response was assessed. Following treatment of 3D PT tissues daily for 7 days with vehicle or TGFβ, viability was measured by resazurin reduction and the expression of genes associated with human renal tubulointerstitial fibrosis was measured. No significant loss of metabolic activity was detected in tissues treated with TGFβ compared to tissues treated with vehicle (Figure 10A). At a dose of 3.3 and 10 ng/ml TGFβ, a reduction in GGT activity was observed, indicating that the higher doses may impact the function of epithelial cells or induce toxicity (Supplemental Figure 3B). TGFβ elicited a dose-dependent increase in collagen 1 (COL1A1), connective tissue growth factor (CTGF), fibroblast-activating protein (FAP), and platelet-derived growth factor receptor β (Figure 10B). The fibrotic response of tissues treated with TGFβ was observed histologically by Gomori's trichrome stain for ECM deposition, with TGFβ inducing thickening of the tissue via expansion of the interstitial layer (Figure 10C). The increased deposition of ECM seen by histology was quantified by Sirius Red/Fast Green staining; treatment with TGFβ at 1.1 ng/ml, 3.3 ng/ml, and 10 ng/ml significantly induced collagen levels in 3D PT tissues (Figure 10D). Together, this data highlights the ability of the tissues to manifest the effects of test agents on multiple cell types within the tubule interstitium as well as the epithelium, and may provide a system to assess the multiple pathways leading to renal fibrosis.

## Discussion

To date, very few systems have been developed to study the human renal tubulointerstitial interface *in vitro*. A variety of systems for 3D culture of RPTEC in isolation have been developed, including culturing cells in Matrigel, culturing cells as organoids on a variety of scaffolds such as hyaluronic acid or silk, and culture of RPTEC in microfluidic devices (“kidney on a chip”) (Joraku et al., 2009; Subramanian et al., 2010; Astashkina et al., 2012; Jang et al., 2013). However, these systems lack direct contact between the epithelium and relevant interstitial cell types, including fibroblasts and endothelial cells, that play both a structural role in orienting the epithelium as well as providing a source of growth factors critical for the continued health and organization of the epithelium (Lemley and Kriz, 1991; Kaissling and Le Hir, 2008; Meran and Steadman, 2011). Without these supportive cell types, RPTEC rapidly lose their native phenotype in culture, thus preventing the ability to perform the chronic, low dose exposure studies necessary to predict how a molecule will perform in the clinic. In addition, the lack of supporting interstitial cell types in other culture systems precludes the ability to model drug-induced and disease relevant states that require an interaction between the epithelium and interstitium, like fibrosis.

One goal of this study was to use 3D bioprinting to build and characterize a model in which a renal interstitium supported the continued growth and maintenance of healthy epithelia. The renal fibroblasts and endothelial cells provided a robust source of endogenously-produced extracellular matrix, which enabled tissue formation without the use of exogenous scaffolding as well as supported the formation of open networks of endothelial cells in the interstitial layer and a collagen-rich basement membrane underlying the epithelium. The endothelial networks form open spaces in the interstitium that may allow better access of media and nutrients to the entirety of the tissue. While the interstitial layer is thicker than the native human renal interstitium, the combination of the renal fibroblasts with the endothelial cells does enable a cellular density more reminiscent of the *in vivo* tissue, which contains fibroblast-like cells immediately adjacent to the epithelium (Lemley and Kriz, 1991).

3D PT tissues were evaluated for their ability to recapitulate physiologically-relevant aspects of the *in vivo* proximal tubule, including reconstitution of the intrarenal RAS and barrier functions. The human PT expresses ACE at the apical surface of RPTEC in order to convert angiotensin I to angiotensin II (Schulz et al., 1988; Ichihara et al., 2004), which then plays a critical role in regulating sodium transport to influence hypertension through feedback onto the renal microvasculature and glomerulus (Kobori et al., 2007). The 3D PT model was able to demonstrate angiotensin II conversion in response to angiotensin I stimulation (Figure 3B). Future experiments exploring the RAS in the 3D PT model could potentially be used to separate the effects of new therapeutics for hypertension on the glomerulus vs. the PT, particularly with regard to mitigating nephrotoxicity as a result of hypertension. Another important function of the PT is to serve as an epithelial barrier controlling the movement of specific types of molecules across the monolayer. The PT is the primary site of re-uptake of water and solutes following glomerular filtration, and as such, must form a more permeable barrier than that observed more distally in the nephron (Ussing et al., 1974; Greger, 1996). Monolayer cultures of renal epithelia such as HK-2 and primary RPTEC cells have been shown to exhibit high TEER values of 100–1,000 Ω^*^cm^2^, with a high degree of variability in those values attributed to differences in culture methods and variations between human kidney donors (Prozialeck et al., 2006). In contrast, *in vivo* tubules exhibit values between 6.6 and 11.6 Ω^*^cm^2^ (Boulpaep and Seely, 1971; Liang et al., 1999). In this study, 3D PT tissues exhibited TEER values of 18.1 Ω^*^cm^2^ (Table 3), which more closely matches values measured *in vivo*. Monolayer epithelial cultures with tight barrier function and high TEER values (>100 Ω^*^cm2) exhibit a P_app_ of 0.5- 1 × 10^−6^ cm/s for Lucifer yellow (Tran et al., 2004). The average P_app_ value for Lucifer yellow in 3D PT tissues was 6.31 × 10^−6^ cm/s for A→B and 5.33 × 10^−6^ cm/s for B→A, indicating transcellular or paracellular transport through the tissues and confirming the leaky barrier function noted by TEER measurements (Table 3). One possible cause for this is the presence of the extracellular matrix-rich interstitium underlying the RPTEC, which may support the formation of a leaky barrier through formation of a physiologically-relevant basement membrane structure. It will be important to evaluate additional substrates for apparent permeability in this system, as previous reports in rat and killifish culture systems have indicated that Lucifer yellow may undergo active transport in the proximal tubules of these non-human species (Masereeuw et al., 1999).

Primary human RPTEC provide the advantage of expressing a variety of transporters known to play a role in drug-induced kidney injury; however, these cells can be cultured for a limited time (<14 days) before undergoing senescence or epithelial to mesenchymal transition and concomitant loss of renal transporter expression and function (Supplemental Figure 2). In contrast, culturing these cells in a 3D context on an interstitial layer enabled retention of epithelial cell viability and function for at least 30 days in culture while retaining gene expression of many renal transporters such as cubilin and megalin, MATE1 and MATE2K, OCT2, BCRP, and P-gp (Supplemental Table 1). Of particular note, the key xenobiotic transporters OAT1 and OAT3 exhibited low or non-detectable levels of mRNA in all the donors tested. In the liver, a discrepancy between mRNA and protein levels has been characterized for hepatic transporters, with most transporters evaluated showing no correlation between mRNA expression by qPCR and protein expression detected my LC-MS/MS (Ohtsuki et al., 2012). Following detection of low amounts of OAT1 and OAT3 mRNA, we evaluated the expression of P-gp, OCT2, OAT1, and OAT3 by LC-MS/MS. For the 4 transporters evaluated, 3D PT tissues exhibited comparable levels of expression to that observed for normal human kidney cortical tissue (Figure 4). While this method enabled comparison of 3D PT tissues and human kidney for each transporter, absolute quantitative values were not able to be determined, which may also influence transporter functional activity. To confirm functional transport, the activity of the xenobiotic transporter P-gp and endogenous substrate transporter SGLT2 were confirmed in 3D PT tissues by transport of R123 and a glucose analog, respectively (Figures 5, 6). LC-MS/MS detection of SGLT2 was hampered by a lack of optimized detection methods, but stable SGLT2 expression was detected by qPCR (Supplemental Table 1), correlating with the functional activity observed. Rhodamine 123 has previously been used to demonstrate P-gp function in HK-2 cells (Tramonti et al., 2009); however, in isolated rat kidneys, additional renal transporters such as OCT1 and OCT2 were involved in rhodamine 123 transport (Heemskerk et al., 2008). Future studies will investigate the function of renal transporters by assessing the directional transport of small molecules coupled with detection by LC-MS/MS, using specific substrates and inhibitors. The ability to do this in a native expression system may enable more accurate prediction of active transport potential, decreasing the reliance on artificial overexpression models. Sustained expression and function of renal transporters in the 3D PT tissues may also enable the elucidation of complex molecular mechanisms of action of human nephrotoxins following chronic exposure.

A human 3D multi-cellular renal tissue composed of distinct epithelial and interstitial cell compartments provides a unique test platform for evaluating new drug entities for potential nephrotoxicity, allowing for the assessment of biochemical, transcriptional, and histological endpoints across multiple cell types and anatomical locations *ex vivo*. To provide initial proof-of-concept data that this model may be used for nephrotoxicity testing, 3D PT tissues were exposed to the classical nephrotoxin cisplatin. 3D PT tissues exhibited an LD50 value of 5.72 μM (Figure 7A), consistent with previously reported values for *in vitro* and *ex vivo* cisplatin toxicity (Tay et al., 1988; Katsuda et al., 2010). In addition, cisplatin treatment led to a loss of epithelial cells in the tissue, as assessed by histology. While several mechanisms likely play a role in cisplatin-mediated nephrotoxicity, including generation of reactive oxygen species and creation of toxic intermediates through glutathione conjugation, these mechanisms occur after cisplatin has been taken up by RPTECs (Hanigan and Devarajan, 2003). This uptake is thought to occur primarily through the action of the OCT2 renal transporter, although other transporters such as the copper transporters (CTR1 and 2) may play a role as well (Ciarimboli et al., 2005). In the current study, inhibition of the OCT2 transporter by cimetidine successfully protected against cisplatin-induced loss of viability and epithelial function (Figures 7–9). This mechanism is clinically relevant, as polymorphisms in OCT2 that influence its function are predictive of cisplatin-induced AKI, and animal models that lack OCT2 expression exhibit decreased sensitivity to cisplatin (Ciarimboli et al., 2005, 2010). In response to apoptosis and loss of PT epithelial cells during AKI, the PT epithelium has demonstrated a high capacity for compensatory proliferation and repopulation *in vivo* (Fujigaki, 2012). Analogously, we observed a dose-dependent increase in proliferating RPTEC in 3D PT tissues exposed to cisplatin, which was reduced in tissues treated with cimetidine (Figure 9). In humans, cimetidine therapy or the presence of loss-of-function mutations in OCT2 correlated with decreased urinary cystatin C following cisplatin administration, demonstrating the possible utility of this therapy as an ameliorative during chemotherapy (Zhang and Zhou, 2012).

Following validation of basic PT-relevant characteristics and the ability to respond to the nephrotoxin cisplatin, we next sought to demonstrate a feature of this novel model that could not be demonstrated by traditional epithelial monolayer cultures. To demonstrate aspects of tubulointerstitial fibrosis that required interaction between the PT epithelial cells and the interstitial cells of the model, we treated 3D tissues with TGFβ, a peptide growth factor required for many aspects of cellular homeostasis including intracellular signaling, proliferation, apoptosis, and ECM deposition. In the kidney, TGFβ signaling, often downstream of kidney injury, plays a role in induction of a fibrotic response, characterized by epithelial to mesenchymal transition, expansion of myofibroblasts, increased immune infiltration, and excessive ECM deposition (Meran and Steadman, 2011; Moll et al., 2013). Following treatment of 3D PT tissues with TGFβ for 7 days, we observed an increase in fibrosis-related genes as well as a dramatic increase in tissue thickness and ECM deposition as demonstrated histologically (Figure 10). The increase in expression of *COL1A1, CTGF, FAP*, and *PDGFRB* have been previously shown to correlate with human tubulointerstitial fibrosis (Henger et al., 2004; Meran and Steadman, 2011; Nogare et al., 2013; Ostendorf et al., 2014) and were confirmed in this system. Loss of GGT activity was observed at the highest dose of TGFβ tested (10 ng/ml) (Supplemental Figure 3C) without an overt loss of metabolic activity (Figure 10A). One possibility is that the PT cells of the 3D PT tissues are undergoing a TGFβ-induced epithelial-to-mesenchymal transition, although this hypothesis remains to be validated. Future experiments will be directed at evaluating drug-induced renal fibrosis using this system, with the goal of developing novel anti-fibrotic drugs to ameliorate this condition, which can lead to loss of kidney function.

In summary, we have designed and validated a new *in vitro* human 3D tissue model capable of preserving RPTEC function over an extended time in culture and enabling quantitative detection of PT nephrotoxicity occurring by specific mechanisms in multiple cell types. These data suggest that 3D PT tissues could positively impact the pre-clinical drug discovery pipeline, helping to prevent costly failures in late stage clinical trials. The inclusion of a tubulointerstitial interface in the model allows for exploration of complex, multifactorial disease processes like fibrosis, as well as assessing the capacity of the RPTEC to compensate, repopulate and/or regenerate during or after drug-induced injury. The importance of developing more complex, multicellular *in vitro* kidney models has also led to the development of stem-cell derived organoids containing multiple regions of the nephron (Takasato et al., 2014, 2015, 2016). While these models display increased architectural and cellular complexity, the differentiated cells retain a more immature fetal phenotype that may limit their utility in toxicology screening (Takasato et al., 2015). In addition, transport studies are difficult to perform in self-organizing organoids due to the restricted access to the tubule lumen. While the region of focus is more limited, the ability to incorporate primary adult epithelial and interstitial cells in the 3D PT model in an architecture that allows access to both the luminal and basolateral surfaces overcomes some of the challenges of renal organoids. Use of primary human RPTECs from multiple donors, including those from patients with acute or chronic kidney disease, may enable better understanding of how drugs may perform clinically across a specific patient population. Additional studies across a panel of nephrotoxic compounds with differing mechanisms of action will help to further elucidate the value of the system for screening new chemical entities. In addition, a single donor of PT epithelial cells was evaluated in this study to provide initial proof-of-concept. Future studies will evaluate the performance of cells isolated from multiple donors, which is critical to fully validate the model. It is also worth noting that the current system is a static culture, which is quite different from the laminar flow that a proximal tubule lumen experiences *in vivo*. The inclusion of laminar flow across the RPTEC could further enhance tubular epithelial cell function; future work will focus on designing a culture apparatus that allows for the inclusion of laminar flow across the apical surface. The system may also enable the parallel investigation of biomarkers that may be useful in noninvasively detecting early kidney injury.

## Author contributions

SK designed, completed, and analyzed experiments and prepared the manuscript. JH, CN, TS, EP, CF, and AD completed experiments and analyzed data. VS completed mass spectrometry analyses. AC, SP, and DN contributed to the conception of the work and in drafting and revising the manuscript.

## Funding

This work was supported by internal funding sources.

### Conflict of interest statement

The authors declare that SK, JH, CN, TS, EP, CF, AD, AC, SP, and DN are employees of Organovo Holdings, Inc. VS is an employee of Ardea Biosciences, Inc.

## Abbreviations

3D: three-dimensional
ACE: angiotensin-converting enzyme
AGT: angiotensinogen
AGTR1: angiotensin receptor type I
AKI: acute kidney injury
DPBS: Dulbecco's phosphate buffered saline
ECM: extracellular matrix
H&E: hematoxylin and eosin
HUVEC: human umbilical vein endothelial cell
LDH: lactate dehydrogenase
OCT: organic cation transporter
P_app_: passive permeability
PCNA: proliferating cell nuclear antigen
PT: proximal tubule
R123: rhodamine 123
RAS: renin-angiotensin system
RFU: relative fluorescence units
RPTEC: renal proximal tubule epithelial cell
TEER: trans-epithelial electrical resistance.
