# Supplementary material for: 3D Proximal Tubule Tissues Recapitulate Key Aspects of Renal Physiology to Enable Nephrotoxicity Testing
Source: Front Physiol. 2017 Mar 8;8:123. doi: 10.3389/fphys.2017.00123 (PMC5340751; doi:10.3389/fphys.2017.00123)
Supplement: Supplementary file 1 [file Table1.PDF]

Target Name	Day 3				Day 12					Day 18					Day 24					Day 30				
	ΔCt	RQ (GOI compared to GAPDH)	RQ (KRT18 compared to GAPDH)	KRT18-normalized RQ	ΔCt	RQ (GOI compared to GAPDH)	RQ (KRT18 compared to GAPDH)	KRT18-normalized RQ	**Fold change relative to day 3**	ΔCt	RQ (GOI compared to GAPDH)	RQ (KRT18 compared to GAPDH)	KRT18-normalized RQ	**Fold change relative to day 3**	ΔCt	RQ (GOI compared to GAPDH)	RQ (KRT18 compared to GAPDH)	KRT18-normalized RQ	**Fold change relative to day 3**	ΔCt	RQ (GOI compared to GAPDH)	RQ (KRT18 compared to GAPDH)	KRT18-normaliz ed RQ	**Fold change relative to day 3**
KRT18	0.93	N/A	0.53	N/A	3.01	N/A	0.12	N/A	**0.23**	3.16	N/A	0.11	N/A	**0.21**	3.22	N/A	0.11	N/A	**0.21**	3.13	N/A	0.11	N/A	**0.22**
ACE	10.8	5.6	0.53	10.81	14.38	0.47	0.12	3.84	**0.36**	13.72	0.79	0.11	6.96	**0.64**	13.77	0.73	0.11	6.65	**0.61**	13.9	0.65	0.11	5.75	**0.53**
AGT	11.71	3	0.53	5.69	12.31	1.97	0.12	15.83	**2.78**	11.83	2.76	0.11	25.03	**4.4**	11.85	2.7	0.11	25.61	**4.5**	11.18	4.32	0.11	37.96	**6.68**
REN	13.99	0.63	0.53	1.18	17.08	0.07	0.12	0.6	**0.51**	15.65	0.2	0.11	1.84	**1.56**	15.33	0.27	0.11	2.37	**2.02**	16.05	0.17	0.11	1.6	**1.36**
ABCB1 (MDR1, P-gp)	12.76	1.44	0.53	2.77	12.07	2.33	0.12	18.91	**6.82**	10.48	7.05	0.11	63.79	**23.02**	11.11	4.7	0.11	42.18	**15.22**	10.17	8.72	0.11	77.6	**28**
ABCG2 (BCRP)	10.93	5.28	0.53	9.82	15.73	0.18	0.12	1.58	**0.16**	12.86	1.71	0.11	15.94	**1.62**	14.77	0.36	0.11	2.77	**0.28**	17.7	0.09	0.11	0.84	**0.09**
AQP1	11.88	2.73	0.53	5.42	14.01	0.6	0.12	4.89	**0.9**	14.01	0.6	0.11	5.44	**1**	14.27	0.51	0.11	4.72	**0.87**	13.39	0.93	0.11	8.21	**1.52**
CUBN	15.45	0.35	0.53	0.63	12.87	1.33	0.12	10.74	**17**	12.55	1.67	0.11	15.03	**23.79**	13.01	1.23	0.11	11.29	**17.88**	12.2	2.19	0.11	21.13	**33.46**
LRP2 (megalin)	14.49	0.44	0.53	0.83	17.13	0.08	0.12	0.69	**0.83**	15.45	0.23	0.11	2.07	**2.5**	16.63	0.1	0.11	0.92	**1.12**	17.3	0.06	0.11	0.54	**0.66**
SLC22A2 (OCT2)	11.24	4.13	0.53	8	12.42	1.83	0.12	14.71	**1.84**	11.99	2.47	0.11	22.09	**2.76**	13.74	0.78	0.11	6.91	**0.86**	12.7	1.6	0.11	14.45	**1.81**
SLC22A6	ND	ND	0.53	ND	17.15	0.07	0.12	ND	**ND**	17.3	0.06	0.11	ND	**ND**	ND	ND	0.11	ND	**ND**	ND	ND	0.11	ND	**ND**
SLC22A8 (OAT3)	16.17	0.14	0.53	ND	ND	ND	0.12	ND	**ND**	17.67	0.05	0.11	ND	**ND**	ND	ND	0.11	ND	**ND**	ND	ND	0.11	ND	**ND**
SLC47A1 (MATE1)	12.31	2.04	0.53	3.78	14.5	0.43	0.12	3.52	**0.93**	14.43	0.52	0.11	4.85	**1.28**	15.73	0.19	0.11	1.87	**0.5**	16.47	0.19	0.11	1.55	**0.41**
SLC47A2 (MATE2K)	15.43	0.44	0.53	0.79	14.21	0.6	0.12	4.69	**5.98**	15.41	0.24	0.11	2.15	**2.74**	15.34	0.26	0.11	2.31	**2.94**	14.02	0.6	0.11	4.88	**6.21**
SLC5A2 (SGLT2)	ND	ND	0.53	ND	16.38	0.12	0.12	0.88	**ND**	17.15	0.07	0.11	0.63	**0.71**	15.93	0.191	0.11	2.03	**2.3**	15.07	0.36	0.11	3.02	**3.43**
NAPT2C	ND	ND	0.53	ND	18.75	0.024	0.12	0.2	**ND**	18.12	0.036	0.11	0.33	**1.55**	18.66	0.025	0.11	0.23	**1.05**	18.09	0.036	0.11	0.33	**1.52**
NHE3	ND	ND	0.53	ND	17.96	0.042	0.12	0.21	**ND**	16.64	0.091	0.11	0.83	**2.35**	16.94	0.082	0.11	0.75	**1.97**	15.91	0.16	0.11	1.45	**3.89**

Supplemental Table 1. Complete gene expression data for 3D PT tissues over 30 days in culture. Data shown is the average of 2 tissue samples per time point. For each gene, data shown is the delta Ct compared to GAPDH, the relative quantitation (RQ) compared to GAPDH, RQ for KRT18 compared to GAPDH, the KRT18-normalized RQ for each gene, and the fold change relative to the day 3 sample. N/A, not applicable. ND, not detected.
