# Supplementary figures and images for: 3D Proximal Tubule Tissues Recapitulate Key Aspects of Renal Physiology to Enable Nephrotoxicity Testing
Source: Front Physiol. 2017 Mar 8;8:123. doi: 10.3389/fphys.2017.00123 (PMC5340751; doi:10.3389/fphys.2017.00123)

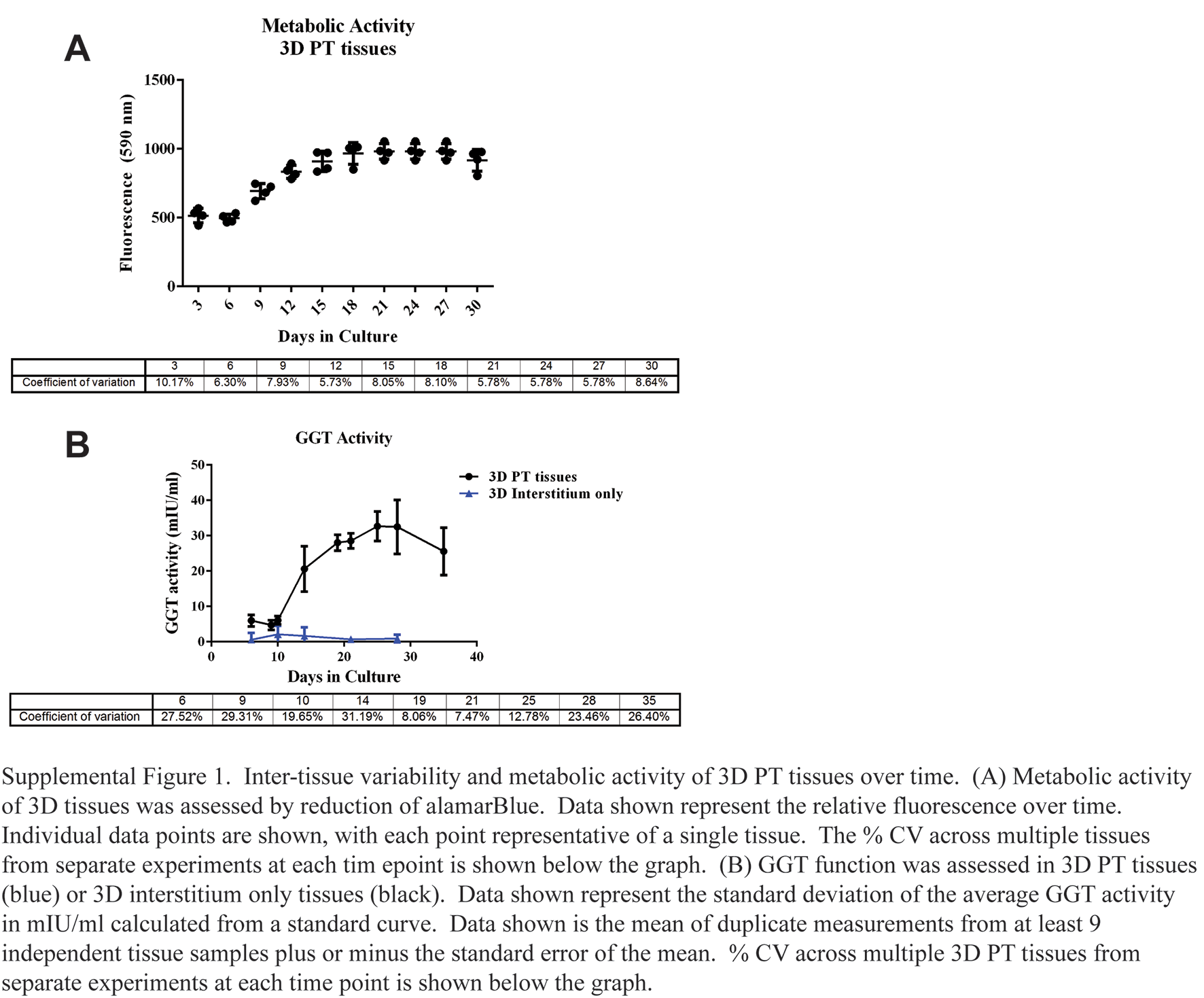

Supplement: Supplementary file 2 [file Image1.TIF]

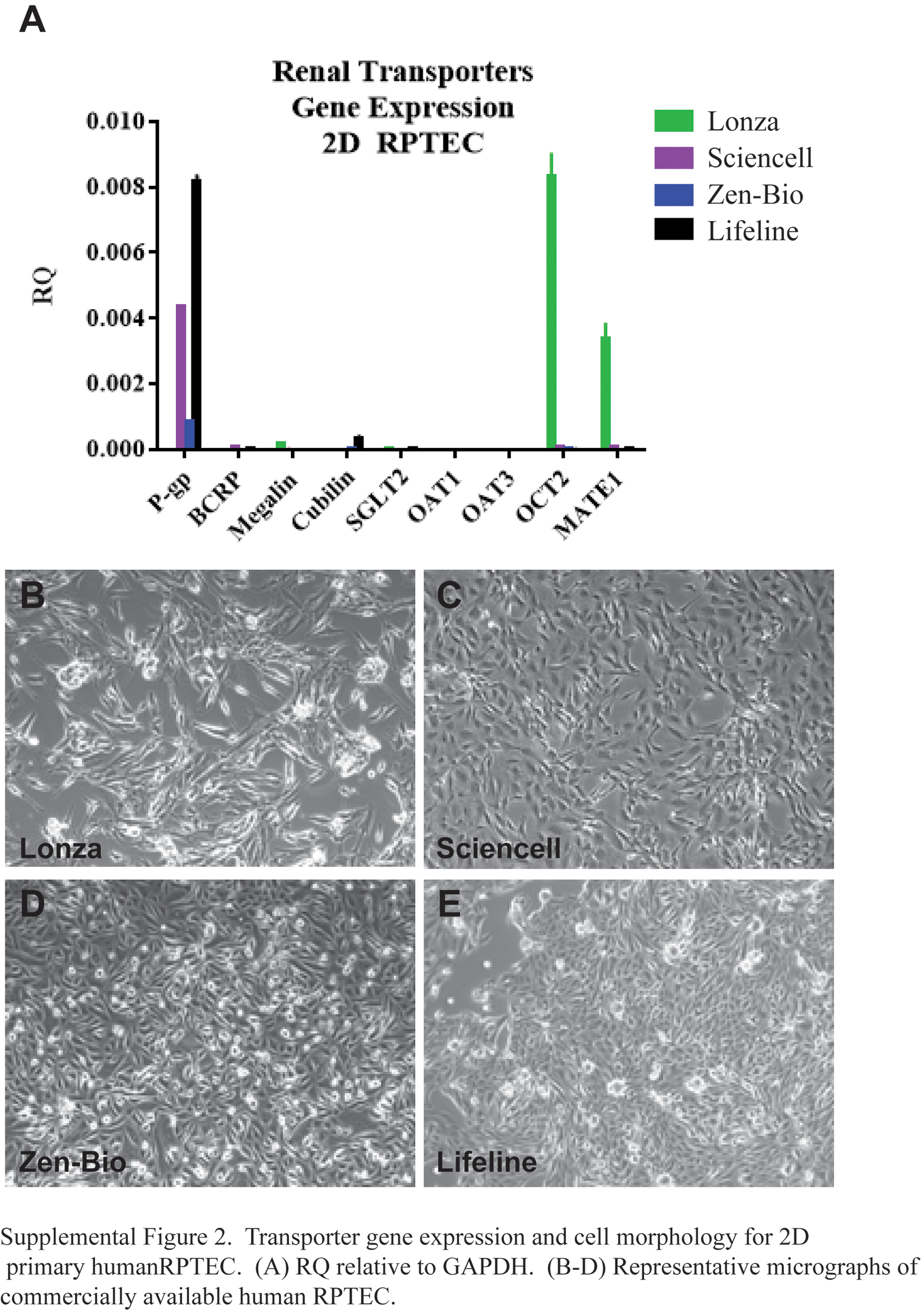

Supplement: Supplementary file 3 [file Image2.TIF]

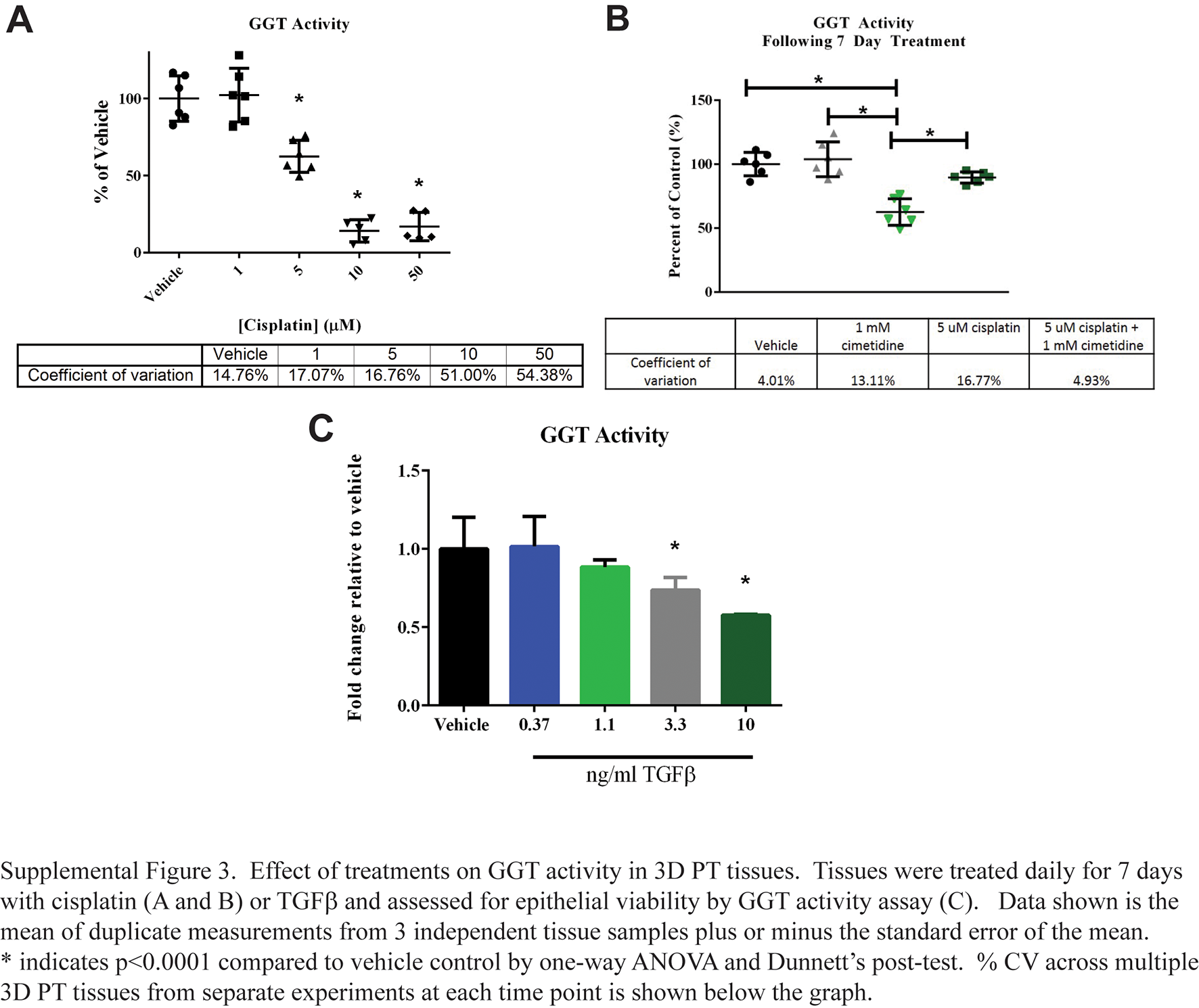

Supplement: Supplementary file 4 [file Image3.TIF]
